# Alzheimer’s disease and its treatment–yesterday, today, and tomorrow

**DOI:** 10.3389/fphar.2024.1399121

**Published:** 2024-05-24

**Authors:** A. Y. Kim, S. Al Jerdi, R. MacDonald, C. R. Triggle

**Affiliations:** ^1^ Medical Education, Weill Cornell Medicine—Qatar, Doha, Qatar; ^2^ Department of Neurology and Medical Education, Weill Cornell Medicine—Qatar, Doha, Qatar; ^3^ Health Sciences Library, Weill Cornell Medicine—Qatar, Doha, Qatar; ^4^ Department of Pharmacology and Medical Education, Weill Cornell Medicine—Qatar, Doha, Qatar

**Keywords:** Alzheimer’s disease, acetylcholinesterase inhibitors, donepezil, N-methyl-Daspartate receptor, memantine, amyloid protein, monoclonal antibody, lecanemab

## Abstract

Alois Alzheimer described the first patient with Alzheimer’s disease (AD) in 1907 and today AD is the most frequently diagnosed of dementias. AD is a multi-factorial neurodegenerative disorder with familial, life style and comorbidity influences impacting a global population of more than 47 million with a projected escalation by 2050 to exceed 130 million. In the USA the AD demographic encompasses approximately six million individuals, expected to increase to surpass 13 million by 2050, and the antecedent phase of AD, recognized as mild cognitive impairment (MCI), involves nearly 12 million individuals. The economic outlay for the management of AD and AD-related cognitive decline is estimated at approximately 355 billion USD. In addition, the intensifying prevalence of AD cases in countries with modest to intermediate income countries further enhances the urgency for more therapeutically and cost-effective treatments and for improving the quality of life for patients and their families. This narrative review evaluates the pathophysiological basis of AD with an initial focus on the therapeutic efficacy and limitations of the existing drugs that provide symptomatic relief: acetylcholinesterase inhibitors (AChEI) donepezil, galantamine, rivastigmine, and the N-methyl-D-aspartate receptor (NMDA) receptor allosteric modulator, memantine. The hypothesis that amyloid-β (Aβ) and tau are appropriate targets for drugs and have the potential to halt the progress of AD is critically analyzed with a particular focus on clinical trial data with anti-Aβ monoclonal antibodies (MABs), namely, aducanumab, lecanemab and donanemab. This review challenges the dogma that targeting Aβ will benefit the majority of subjects with AD that the anti-Aβ MABs are unlikely to be the “magic bullet”. A comparison of the benefits and disadvantages of the different classes of drugs forms the basis for determining new directions for research and alternative drug targets that are undergoing pre-clinical and clinical assessments. In addition, we discuss and stress the importance of the treatment of the co-morbidities, including hypertension, diabetes, obesity and depression that are known to increase the risk of developing AD.

## 1 Introduction

Dementia is a multifaceted neurological disorder that covers a spectrum of cognitive impairments primary affecting memory, thinking, attention, behavior, and the ability to perform everyday tasks. Demographically, it disproportionally affects older adults, with the risk significantly increasing after the age of 65. While various types of dementia exist, such as vascular dementia, including CADASIL (cerebral autosomal dominant arteriopathy with subcortical infarcts and leukoencephalopathy), the Notch 3 gene mutation that manifests as cerebral arteriopathy, Lewy body dementia, and frontotemporal dementia, it is Alzheimer’s Disease (AD) stands out as the most prevalent form, accounting for approximately 60% of all dementia cases. Despite the diversity among dementia subtypes, AD is the primary culprit, imposing profound emotional and economic burdens on individuals, families, and healthcare systems worldwide, especially in rapidly aging populations. As such, as reflected in Supplementary Figure S1, the search for the cause(s) and effective treatment(s) of AD has intensified over the years since its first description by Alois Alzheimer in 1907 (Alzheimer, 1907; Alzheimer et al., 1995, also see Alzheimer’s, 2020).

A major difficulty in developing effective therapeutic agents is linked to the long pre-clinical phase between the early stages of the brain pathology and the detection of cognitive decline (McGeer, et al., 2018). Clinically, AD has both a familial (FAD) and a sporadic (SAD) occurrence, with FAD accounting for approximately 5% of cases (Ertekin-Taner, 2007). Given these complexities, it is not surprising that the determination of the most effective treatment for AD as well as optimizing and providing early treatment for individuals with AD has proved difficult–a challenge that to date has not been met (Ferrari and Sorbi, 2021).

AD can be distinguished from other types of dementia by its closer association in its early stage with short term memory impairment, and, later by a buildup of amyloid protein (Aβ) in the brain. The greater majority of cases of AD are seen in subjects over the age of 65 and referred to as Late Onset AD (LOAD), or sporadic AD (SAD); however, approximately 5%–10% of cases are seen in younger patients and referred to as Early Onset AD (EOAD), or familial AD (FAD, FD), which is seen in patients as young as 30 years of age and may have an atypical clinical presentation (Sirkis et al., 2022). Unlike LOAD patients, EOAD has a strong genetic determination and autosomal-dominant inheritance linked primarily to three genes: Amyloid Precursor Protein (APP), Presenilin-1 (PSEN1), and Presenilin-2 (PSEN2), which code for presenilin-1 (PS-1), and presenilin-2 (PS-2) respectively, with mutations in PSEN1 being more frequent (Cacace et al., 2016). Presenilin are a family of transmembrane proteins that make up the catalytic component of γ-secretase, an enzyme which cleaves more than 140 substrates, including APP (Bagaria, et al., 2022; Hur, 2022)–see section 3.3. However, as a further complication to the understanding the pathophysiological basis of AD and effective treatment(s), genome-wide association studies (GWAS) have identified over 50 loci linked to AD and notably associated with three genes: the clusterin (CLU) gene; the PICALM (phosphatidylinositol-binding clathrin assembly) gene; and the complement component (3b/4b) receptor one on chromosome 1 (CR1) (reviewed by Sims et al., 2020). EOAD also has a nonmendelian link to the apolipoprotein E4, ApoE4, allele (Reitz et al., 2020), with polymorphisms associated with lipid metabolism, immunity and endocytosis pathways that contribute to sporadic AD (Barber et al., 2017), and offer additional targets for optimizing the treatment of AD (reviewed by Femminella et al. (2021)–see also section 3.5.

**FIGURE 1:**
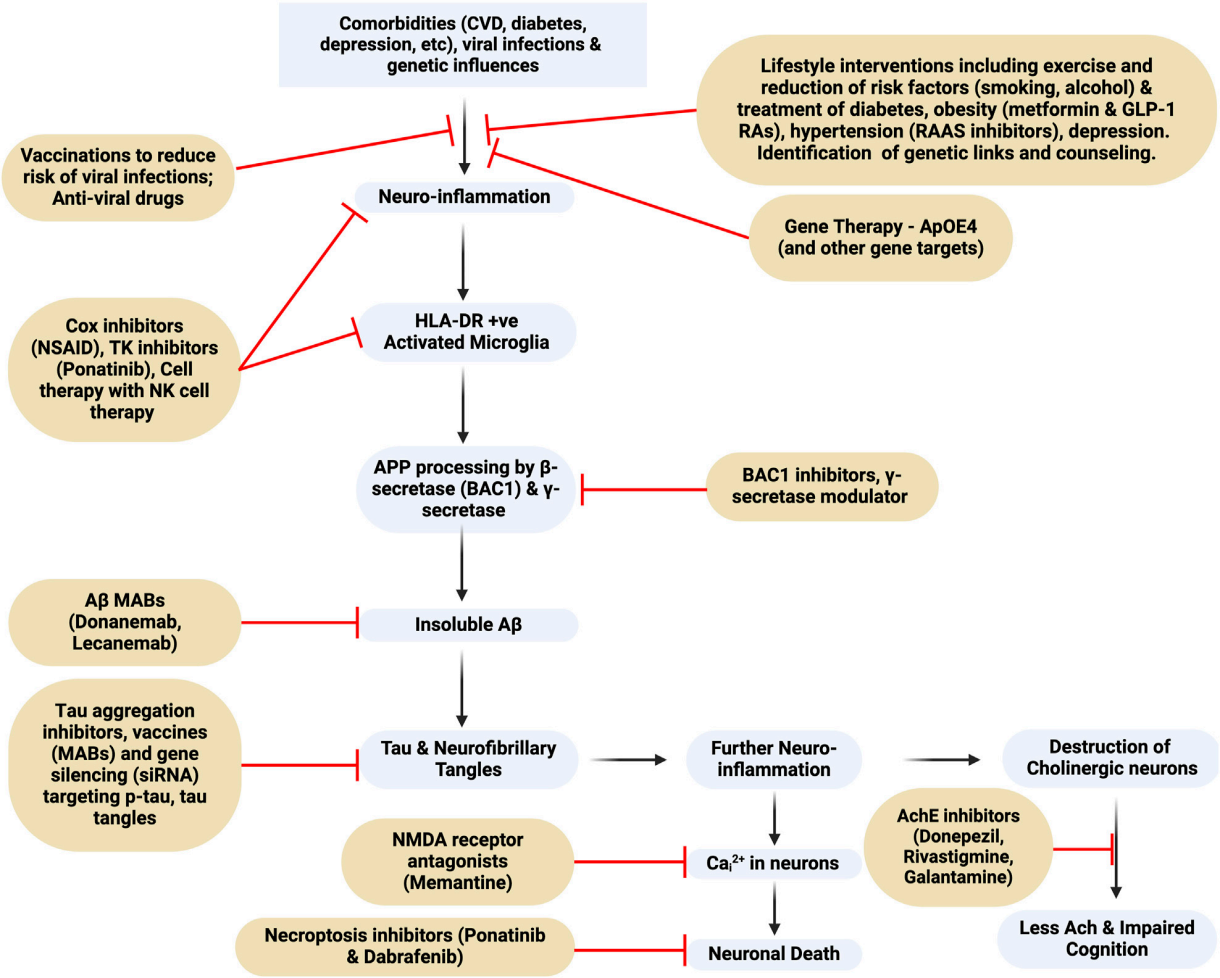
Flowchart of Alzheimer’s Dementia pathogenesis and targets for drug intervention. MAB, monoclonal antibody; BAC1, beta-secretase 1; NMDA-receptor, N-Methyl-D-Aspartate receptor; TK, tyrosine kinase; NK, natural killer. This figure was created with BioRender.com.

An analysis of data from the UK biobank of over 350,000 participants aged under 65 with dementia identified 39 risk factors that included not only CVD, diabetes and depression, but also vitamin D deficiency, benzodiazepine and alcohol use, smoking, a low level of physical activity, lower grip strength, environmental factors, depression, and sleep problems (Hendriks et al., 2023). These data, as also reflected in Figure 1, emphasize the importance of a greater focus on identifying the key modifiable risk factors that play an important role in both EOAD and LOAD to prevent disease development, as was stressed in the 2020 Lancet Commission report (Livingston et al., 2020). The importance of reducing these modifiable risk factors has been emphasized by many others (see also Wang et al., 2018; Zhang et al., 2021).

In very rare instances AD may result from a prion-like transfer of Aβ that seeds the buildup of plaques, as has been reported in 8 cases and linked to the subjects being treated with cadaver-derived growth hormone (Banerjee, et al., 2024). These cases complement earlier evidence of a prion-like transfer (Jaunmuktane et al., 2015; Purro et al., 2018), and was also the title of a 1984 article in the New England Journal of Medicine ‘Some speculations about prions, amyloid, and Alzheimer’s disease’ (Prusiner, 1984). Collectively, these findings suggest that under certain rare circumstances AD is transferable, although confirmation is required (Jucker and Walker, 2024).

Fully effective cures or disease-modifying drugs for AD are lacking and currently approved drugs belong to three classes; 1/acetylcholinesterase inhibitors (AChEIs), 2/NMDA receptor modulators (memantine), and 3/anti-amyloid monoclonal antibodies (anti-Aβ MABs), however, other putative cellular pathways involved in the pathology have been identified, and are summarized in Figure 1. Also included is a brief review of NSAIDs, which may reduce neuroinflammation, anti-diabetes drugs such as metformin and glucagon-like peptide receptor agonists (GLP-1 RAs), and anti-hypertensive drugs, whose effects are to reduce the impact of diabetes and obesity co-morbidities that enhance the risk of developing AD. Recognizing the limitation of the anti-Aβ-MABs other potential targets are discussed including neuroinflammation, which is discussed in the context that a very early stimulus for AD is the activation of microglia. Of historical significance it was McGeer et al. (1987) who first reported the presence of the class II major histocompatibility antigen, HLA-DR, on microglia in the hippocampus and linked HLA-DR to AD and the presence of Aβ plaques as well as a negative correlation with cortical choline acetyltransferase. Later, Mattiace et al. (1990) demonstrated that HLA-DR was constitutively expressed in white matter, but in grey matter it was induced as a result of the disease buildup of Aβ.

### 1.1 Tests for diagnosing AD and determining the effectiveness of drugs

Tests for both the diagnosis and for the determination of the development and effectiveness of drugs are of critical importance for treating AD as well as important aids in determining the effectiveness of drugs. However, there is no single definitive test for the diagnosis of AD, and a combination of diagnostic tools, medical history review, cognitive and functional assessments, as well as brain imaging, cerebrospinal fluid analysis, and blood tests are collectively employed (Schachter and Davis, 2000; Tsoi et al., 2015). The main cognitive and functional tests employed include the Mini-Mental State Examination (MMSE); the Montreal Cognitive Assessment (MoCA); and the Alzheimer’s Disease Assessment Scale–Cognitive Subscale (ADAS-Cog); Addenbrooke’s Cognitive Examination (ACE) A summary of the advantages and limitations of these tests as well key references is provided in Supplementary Table S1. The ADAS-Cog test is designed to specifically measure the cognitive performance of subjects who already carry a diagnosis of AD and is a widely used primary outcome measure in both clinical research and drug trials to track the progression of moderate to severe AD (Rosen et al., 1984). However, ADAS-Cog is not a suitable assessment tool for broader screening or for detecting mild cognitive impairment (MCI) and the subtle cognitive changes associated with the early stages of cognitive decline; in contrast MMSE, MoCA and ACE are broad screening tools that assess a wider range of cognitive functions used in a wide range of dementias (Podhorna et al., 2016).

**FIGURE 2:**
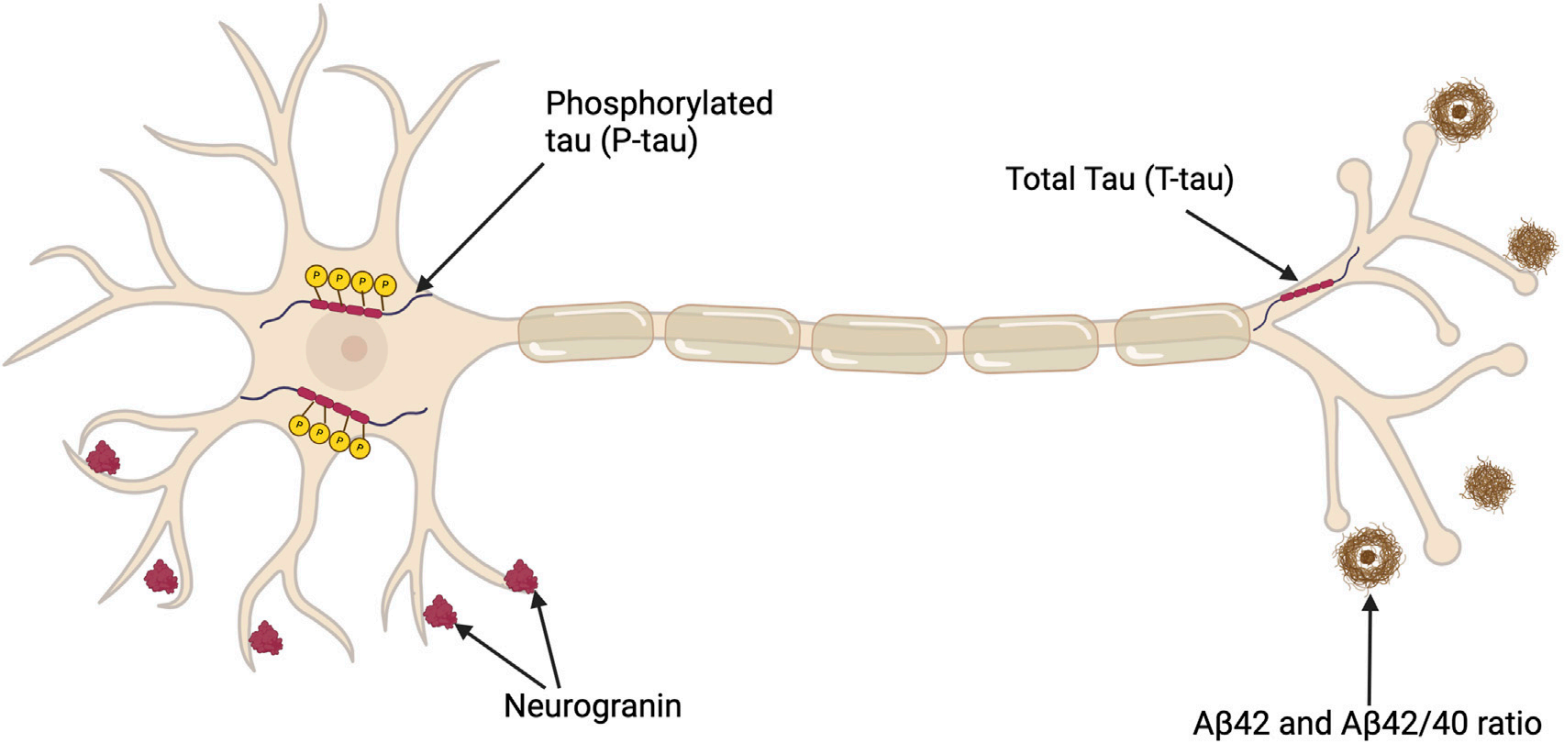
Depiction of a neuron illustrating key CSF biomarkers useful in diagnosing AD in addition to a potential synaptic biomarker candidate, neurogranin. Total tau (T-tau) shows the extent of neuronal damage but lacks specificity for AD. Phosphorylated Tau (P-tau) represents tau proteins with relatively AD-specific modifications. The CSF Aβ42 level is reduced in AD despite the concurrent increase in amyloid (Aβ) deposition in the brain, which is a characteristic of AD pathology. The Aβ42/40 ratio compensates for individual variabilities, thus offering a more standard measure of amyloid pathology. This figure was created with BioRender.com.

Along with cognitive tests, several biomarkers have value in the diagnosis of AD–see Figure 2 and Supplementary Table S2. Biomarkers can be classified as linked to Aβ deposition, pathologic tau and neurodegeneration (Jack et al. (2018). Assessing CSF is a relatively invasive and carries risk, therefore blood biomarkers that could track AD progression would be preferable; however, there are limitations related to quantification and reproducibility as only limited amounts of brain proteins diffuse into the bloodstream (Blennow and Zetterberg, 2015). For instance, although the longitudinal data has indicated an association between elevated plasma tau levels and subsequent cognitive decline, the slight increase in plasma tau may not be diagnostically significant (Mattsson et al., 2016; Blennow and Zetterberg, 2018). However, in a recent study of 786 patients a commercially available kit for p-tau217 provided comparable diagnostic evidence to that obtained from CSF biomarkers (Ashton et al., 2024). Furthermore, an analysis of blood samples in the UK Biobank identified four other plasma proteins linked to AD (Guo et al., 2024). Replication of these findings would prove beneficial to using blood tests to aid in the diagnosis of AD.

In conclusion, current data indicates that CSF biomarkers have a greater diagnostic value than plasma biomarkers; however, improvements in the kits available for assessment of plasma biomarkers may change this conclusion and thereby enhance prospects for earlier diagnosis, assessment of therapeutic treatment and, perhaps, prevention of AD.

## 2 Methods

A critical review of the published literature concerning the treatment of AD was conducted using PubMed and Scopus searches directed at:

(i) The pathophysiological basis of AD as a framework for the identification of drug targets.
(ii) The pharmacological properties of the drugs that have been used to treat AD
(iii) Evaluation of the clinical trial data that supports the use of the different classes of drugs, alone or in combination.
(iv) Discussion of the controversies over the recent approval of monoclonal antibodies directed at beta amyloid plaques.
(v) Discussion of the potential for new targets for drugs, including cell therapy, which are directed at reducing neuroinflammation.

The resulting narrative review paper is supported by over 300 citations that cover the pathophysiological basis for the drugs that have been developed and are currently used to treat AD as well as a critical evaluation of their effectiveness and insights as to other potential targets for new drug development.

This narrative review was the result of searches in the PubMed and Scopus databases databases (see Supplementary Figures S1-4 for search details). Publications were selected based on their study design and use of statistical evidence to support their conclusions.

## 3 Hypotheses and drug targets

Although several hypotheses have been offered to explain the pathophysiological basis for AD it is likely that there are multiple triggers. As of 2019, the largest proportion of clinical trials (23.3%) have addressed the amyloid hypothesis, followed by the neurotransmitter hypothesis (including both acetylcholine and glutamate) (19%), mitochondrial dysfunction (17%), neurovascular (7.9%), exercise (6%), the neuroinflammation hypothesis (4.6%), diabetes (2.3%), links to virus infection (0.5%) (Liu et al., 2019). In this review, we focus on the following hypotheses: (i) cholinergic, (ii) glutamate, (iii) amyloid, (iv) tau, (v) vascular hypothesis, and finally (vi) viruses and the benefit of vaccinations. Interest in the role of virus infections has increased following COVID-19 as many people with “Long COVID” appear to suffer neurological dysfunction and cognitive decline (Venkataramani and Winkler, 2022). Other hypotheses such as those linked to diabetes (de la Monte and Wands, 2008) and mitochondrial dysfunction (Cardoso et al., 2004; Swerdlow et al., 2010) are considered tangentially as linked to the putative benefits of drugs, such as the anti-diabetes drug metformin. For instance, a clinical trial, Metformin in Alzheimer’s Dementia Prevention (MAP), NCT04098666, will be completed in 2026. Metformin has putative effects on mitochondrial function that have been argued to underly a neuroprotective action, and are, at least in part, supported by epidemiological data and human genetic studies linking metformin to the NADH:Ubiquinone Oxidoreductase Subunit A2 (NDUFA2) gene and mitochondrial complex 1 (El-Mir et al., 2008; Campbell et al., 2018; Zheng et al., 2022); however, the contribution of a mitochondrial action of metformin as a basis for its therapeutic benefits have been challenged on the basis of the high concentrations used in in vitro studies (He and Wondisford, 2015; Fontaine, 2018). Other drugs that are used to treat type 2 diabetes (T2D), such as the GLP-1 RAs, also reduce cognitive decline suggesting that the primary benefit of anti-diabetes drugs is via improved glycemic control and reducing the pathophysiological sequalae of metabolic dysregulation, (Nørgaard et al., 2022; Nowell et al., 2023). Epidemiological data also suggests that anti-inflammatory NSAIDs reduce the risk of AD; however, the data is controversial.

### 3.1 Cholinergic hypothesis

**FIGURE 3:**
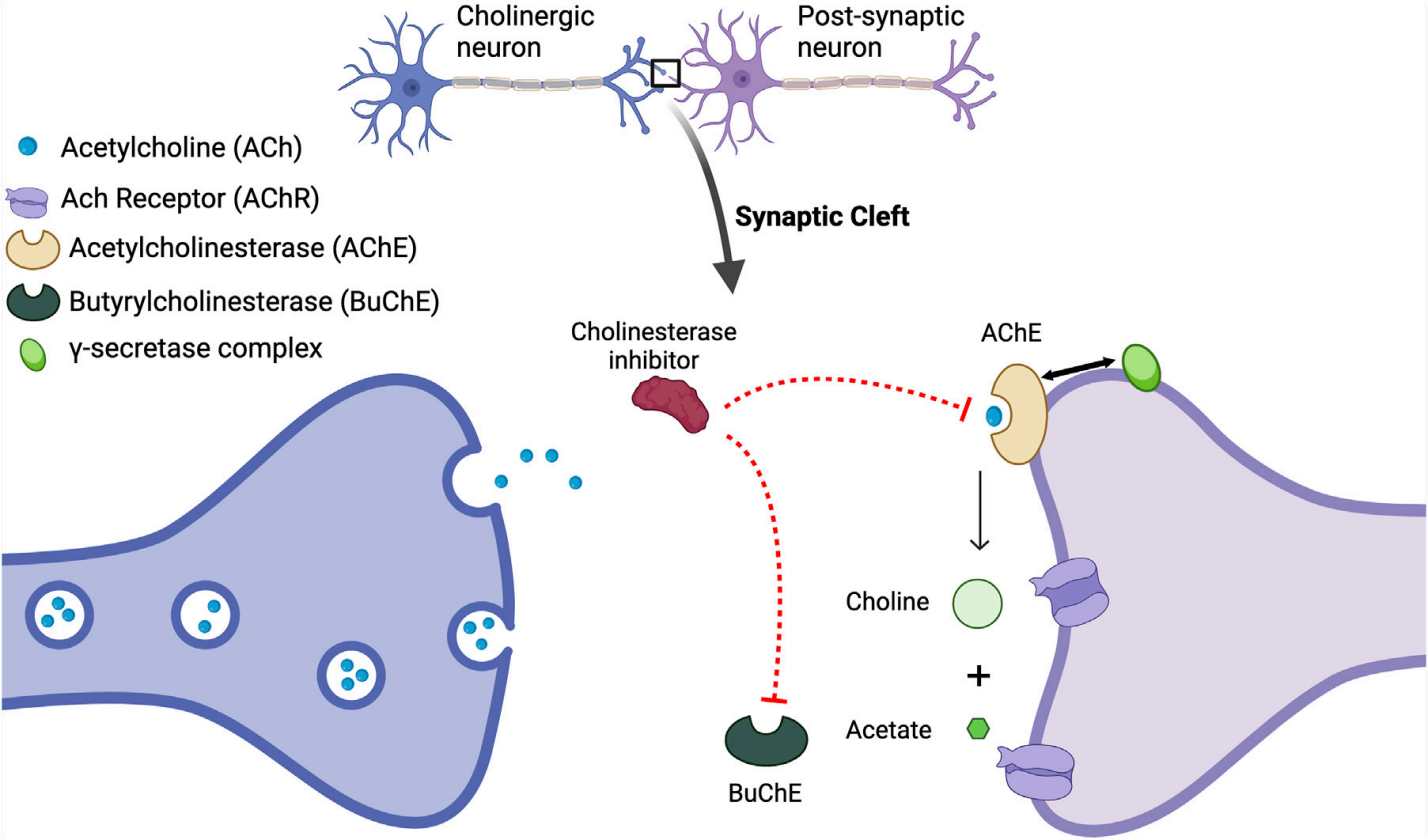
The Acetylcholine Hypothesis and role of AChE Inhibition: Decreased levels of acetylcholine (ACh) contribute to cognitive decline; however, the inhibition of acetylcholinesterase (AChE) with acetylcholinesterase inhibitors (AChEIs) prevents the breakdown of ACh, thus resulting in elevated synaptic ACh levels. The increase in available ACh is associated with improved cognitive function. AChE also interacts with the enzyme presenilin-1 PS-1, which plays a crucial role in Aβ production including the regulation of γ-secretase, and its association with AChE underscores a significant cholinergic-amyloid link in the pathophysiology of AD. Mutations in the PSENI gene result in the enhanced production of Aβ (Silveyra et al., 2008). It has been reported that AChE enhances both transcription and production of PS-1, decreasing γ-secretase activity and reducing the processing of APP (Campanari et al., 2014). Thus, the loss of the regulation of PS-1 by AChE and a subsequent increase in γ-secretase activity will increase Aβ. This figure was created with BioRender.com.

A deficiency of acetylcholine (ACh) in the brain as the pathophysiological basis of AD was first proposed in 1976 and based on the observation that choline acetyltransferase, the enzyme responsible for the synthesis of ACh, was greatly reduced in the amygdala, cortex and hippocampus in postmortem brains from patients with AD compared to brains from non-AD subjects (Davies and Maloney, 1976). Cholinergic neurons, particularly in the basal forebrain, play critical roles in memory, attention, and learning (Whitehouse et al., 1982) and degeneration of ACh-producing neurons in AD patients affects neuronal communication, resulting in memory deficits (Hasselmo, 2006; Chen et al., 2022). As summarized in Figure 3 and based on co-immunoprecipitation data acetylcholinesterase (AChE), the enzyme responsible for the degradation of ACh, binds to and interacts with PS-1 in the same intracellular compartment in CNS neurons (Silveyra et al., 2008).

#### 3.1.1 Enhancing availability of ACh - Acetylcholinesterase inhibitors

The first and the main class of drugs currently used for AD are the cholinesterase inhibitors (AChEI) that have varying specificity for AChEI *versus* pseudo-AChE (also known as butyrl ChE [BuChE]) - see Figure 3, and are widely used for the symptomatic treatment of mild-moderate AD. In the brain, ACh is degraded by both AChE and BuChE (Nordberg et al., 2013), with AChE present in the nerve synaptic junctions, and BuChE in glial cells (Mesulam et al., 2002). In the healthy brain, AChE activity is the main enzyme that breaks down ACh, while in the brains of AD subjects, AChE activity is decreased and BuChE activity is increased to compensate for the reduced AChE activity (Perry et al., 2003). Four AChEIs have been approved for the treatment of AD and have variable specificity for AChE *versus* BuChE. Tacrine was the first to be approved in 1993; however, it was withdrawn in 2013 due to frequent reports of elevated liver enzymes and fatal liver toxicity (Watkins et al., 1994). The three remaining ACEIs are donepezil, rivastigmine, and galantamine. In addition to actions as an AChEI, galantamine also acts as a positive allosteric modulator of nicotinic acetylcholine receptors and potentiates cholinergic neurotransmission (Wang and Reddy, 2017).

All of the currently available AChEIs suffer from the same common gastrointestinal side effects (diarrhea and vomiting) related to their systemic effects on cholinergic transmission, although reportedly less for donepezil (Sugimoto et al., 2000). A summary of the AChEIs, including key references for trial data and meta-analysis is provided in Table 2 together with key information for other drugs that are used to treat AD. Donepezil is the most widely used of the available AChEIs and is used for mild-moderate and severe AD and also combined with the N-methyl-D-aspartate receptor (NMDAR) modulator, memantine (see section, 3.2). Although meta-analysis and several studies conclude that donepezil improves cognitive scores, it is important to realize that AD is a “fluid disease” and measuring cognitive scores for only 24 weeks, or a year, might not be sufficient to accurately determine whether there is slowing of the progression of AD: comparable data from long-term treatment is needed not only for donepezil but all drugs used for AD.

In summary, although widely used for the symptomatic relief of mild-moderate AD, ACHEIs, either alone or in combination with the NMDAR modulator, memantine, have limited effectiveness as disease-modifying drugs. They are not a cure and their use can be associated with troublesome side-effects. A number of trials (see Amenta et al., 2001) have investigated alternative approaches to correcting cholinergic transmission, notably the use of choline precursors including choline and phosphatidylcholine (lecithin) CDP-choline, alphaglyceryl-phosphoryl-choline (α-GPC), choline alphoscerate, and phosphatidylserine. The results from one trial with CDP-choline indicated improved cognitive evaluation scales and arguably slowed the progression of AD (Secades and Frontera, 1995). These data suggest that the use of choline precursors should be re-examined, possibly in combination with AChEIs, to determine whether treatment decreases neuroinflammation and Aβ-associated neurotoxicity as has been shown in pre-clinical studies (Alvarez et al., 1999; Munafò et al., 2024).

### 3.2 Glutamate toxicity hypothesis

**FIGURE 4:**
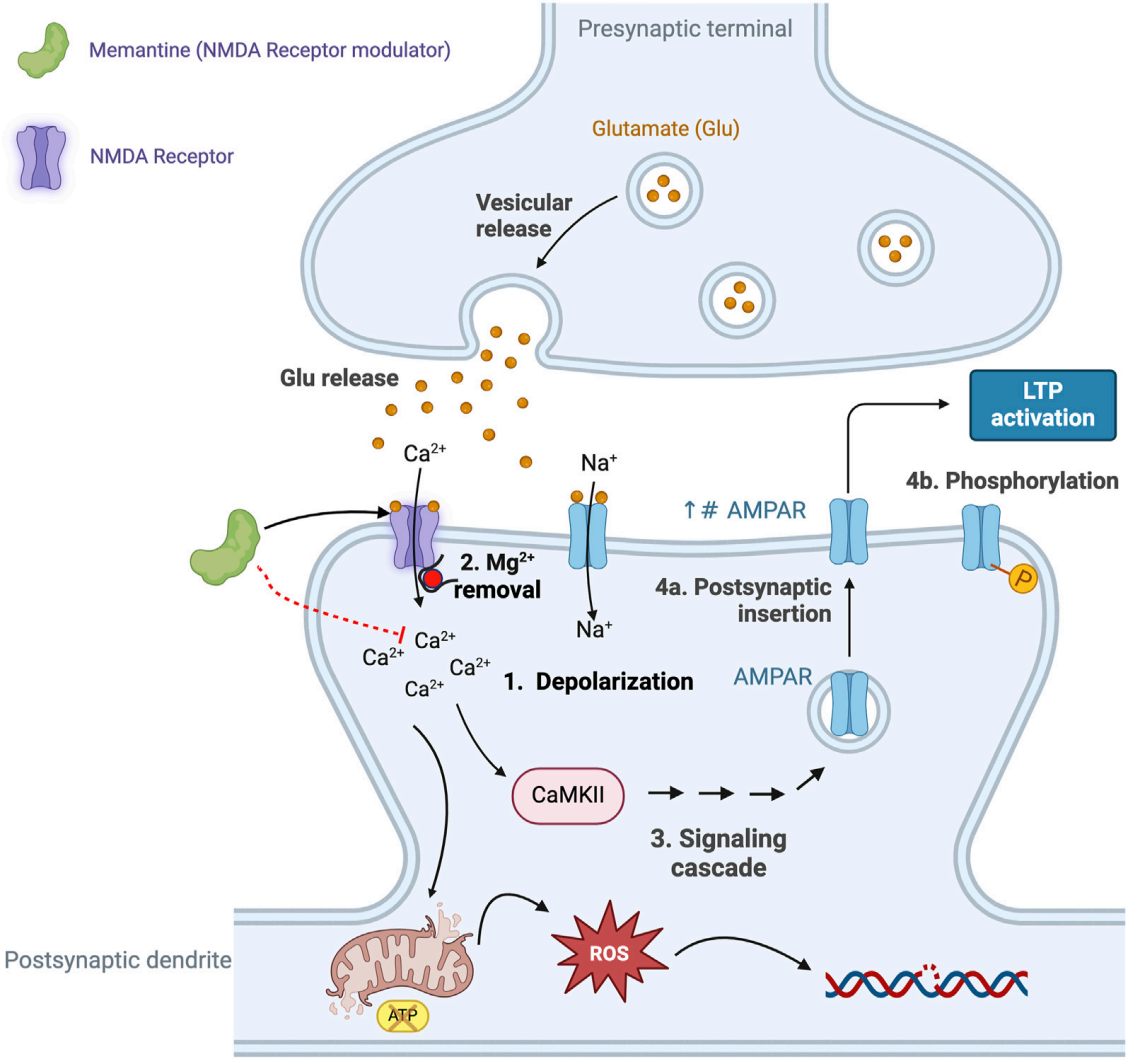
The excitatory neurotransmitter glutamate plays an essential role in synaptic plasticity, a process that refers to the ability of synapses to strengthen, or weaken, in response to neuronal activity. Glutamate-mediated synaptic plasticity involves two key processes: long-term potentiation (LTP) and long-term depression (LTD) (Riedel et al., 2003; Abraham et al., 2019). In brief, LTP requires the activation of α-amino-3-hydroxy-5-methyl-4-isoxazolepropionic acid receptors (AMPAR) and N-methyl-D-aspartate receptors (NMDAR). Activation of the NMDAR is initially prevented due to intracellular Mg2+ blocking the cation channel, however, when glutamate activates the AMPAR this results in the entry of Na+ and depolarization of the neuron (Step 1 in Figure 5) and removal of the Mg2+ block (Step 2 in Figure 5), allowing activation of the NMDAR and the intracellular entry of both Na+ and Ca2+ (Vargas-Caballero and Robinson, 2004; Blanke and VanDongen, 2009). The increase in intracellular Ca2+ initiates a signaling cascade that involves the enzyme calcium/calmodulin-dependent protein kinase II (CaMKII) and triggers the translocation of intracellular AMPARs to the postsynaptic membrane (Step 4a in Figure 4) and phosphorylates AMPAR (Step 4b in Figure 4) (see Sumi and Horada, 2020, for details of the signaling pathway). Collectively, these events result in the strengthening of the neuronal signal and ultimately lead to the development of LTP and memory formation and learning (Wang, 2017), including changes in gene transcription that affect receptor density and result in changes in neuron function. However, the excessive release and presence of glutamate in the neuronal synapse results in elevated levels of intracellular calcium and prolonged cell depolarization (Choi, 1987). This, in turn, results in the generation of increased levels of reactive oxygen species (ROS), causing neuronal damage and cell death (Savolainen et al., 1995). Excess levels of glutamate promote microglia-mediated neuroinflammation, further damaging adjacent neurons (Qin and Crews, 2012; Lee V. M. et al., 2021). This supports the argument that the combination of microglia-mediated inflammation and oxidative stress results in neural damage, synaptic dysfunction and impairment of LTP leading to AD. Elevated Aβ is also associated with hyperactivity of NMDA-mediated currents and neurotoxicity, thus providing a link between the role of Aβ plaques and defective glutaminergic neurotransmission (Harkany et al., 2000; Domingues et al., 2007). This figure was created with BioRender.com.

The glutamate hypothesis is based on evidence that there is a reduction in the binding of l-[3H] glutamate in the postmortem brains of subjects with AD (Greenamyre et al., 1987; Maragos et al., 1987) thus linking a defect in glutaminergic neurotransmission, the principle excitatory pathway in the brain, to AD (Maragos et al., 1987; Hladky and Barrand, 2022). As summarized in Figure 4 glutaminergic transmission and synaptic NMDARs are important for synaptic plasticity, LTP and neuronal survival, and also play an essential role in memory (see Abraham et al., 2019), whereas the activation of extra-synaptic NMDARs is associated with cell death and AD. This association form the basis for the use of the NMDAR antagonist, memantine, for the treatment of AD (Hardingham et al., 2002; Hardingham, 2006; Hardingham and Bading, 2010).

#### 3.2.1 Memantine–A NMDAR antagonist

Memantine was approved by the FDA in 2003 for the treatment of moderate to severe AD and based on the evidence that excessive activation of the NMDAR triggered neuronal toxicity and apoptosis (Zeevalk and Nicklas, 1992; Bonfoco et al., 1995; Thomas and Grossberg, 2009). Memantine binds to the NMDAR in a voltage dependent manner and, since it is a low-affinity uncompetitive antagonist and open-channel blocker, the risk of memantine binding to receptors in the non-depolarized state is low and therefore does not interfere with Long Term Potentiation (LTP) (Bresink et al., 1996). Unlike ketamine, a high affinity NMDAR antagonist acting on allosteric/dizocilpine sites, memantine blocks NMDAR but dissociates rapidly, thus avoiding prolonged receptor blockade and associated negative side effects such as interruption of learning and memory formation processes (Folch et al., 2018). Although memantine has been reported to bind with variable affinity to other sites including dopamine cholinergic, serotoninergic, and also sigma receptors, the contributions of these actions to its therapeutic actions are unknown and it is its action on the NMDAR that is thought to be the major contributor to reducing neuronal excitotoxicity (Seeman et al., 2008).

Although early studies and including meta-analysis indicated that monotherapy with memantine demonstrated greater efficacy than placebo in improving cognitive function a 2019 Cochrane report that included data up to 25 March 2018 from double-blind, placebo-controlled, randomized trials concluded that there was only a small clinical benefit with the use of monotherapy memantine for moderate-severe AD, but not mild-moderate AD (See Table 2; McShane et al., 2019). In terms of safety outcomes, there was no significant difference in all-cause discontinuation between memantine and placebo groups but the memantine-treated group was more likely to develop dizziness (RR = 1.53, 95% CIs = 1.02–2.28, *p* = 0.04). As pointed out in the meta-analysis report by Blanco-Silveste et al. (2018) an effective treatment should have a lower discontinuation rate than placebo as this would indicate that an improvement in symptoms outweighs side effects. Common side effects of memantine are headache, confusion, diarrhea, and constipation.

In summary, despite limitations, questions over effectiveness, and side effects, memantine either alone or in combination with an AChEI, is widely used for the symptomatic treatment for moderate to severe AD.

#### 3.2.2 Combination therapy AChEI plus NMDAR antagonist (donepezil + memantine)

To enhance symptomatic relief a fixed dose combination of memantine ER/donepezil was approved by the FDA in 2014 for patients with moderate to severe AD. Despite contradictory results (see Table 2), the overall conclusion is that combination therapy is more effective than monotherapy in delaying cognitive and functional decline as well as delaying the requirement for nursing home care (see Lopez et al., 2009).

### 3.3 Amyloid hypothesis

**FIGURE 5:**
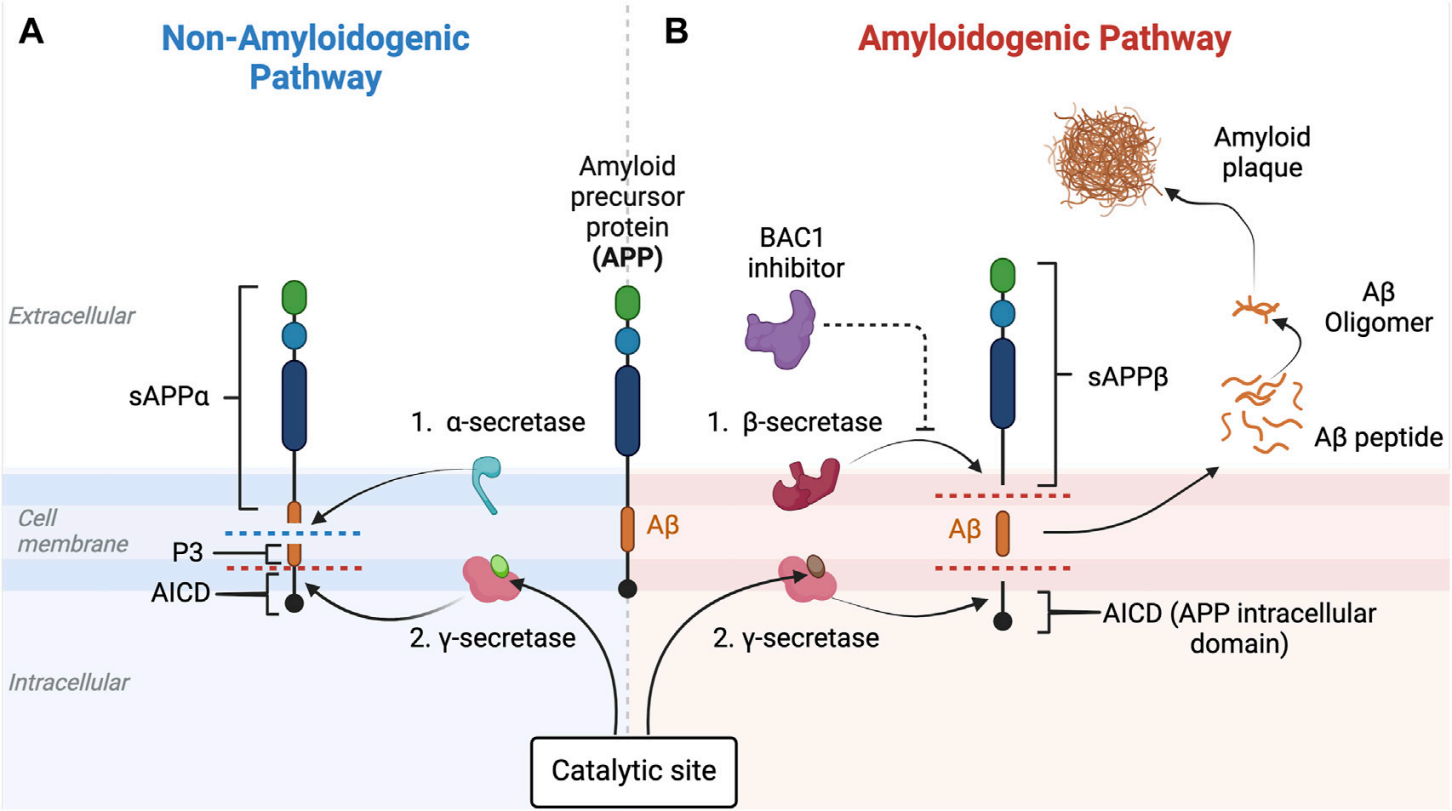
Non-amyloidogenic and amyloidogenic pathways and β-secretase (BACE-1) inhibition. This figure illustrates the non-amyloidogenic **(A)** and amyloidogenic **(B)** pathways of amyloid precursor protein (APP) processing. Light green circle represents normal PSEN-1 protein at the γ-secretase complex. Dark brown circle represents mutated PSEN-1 protein at the γ-secretase complex, which increases production of longer and aggregation-prone beta amyloids. Aβ42 is rich in hydrophobic amino acids such as isoleucine, phenylalanine, and valine. Specifically, the hydrophobic side chains at the positions 41 and 42 increase the propensity of Aβ42 to aggregate (Kim and Hecht, 2005). This promotes the formation of the β-sheet structures characteristic of aggregated amyloid proteins. In a normal physiologic state (Figure 5A), APP is processed by α-secretase and then by β-secretase (Butterfield et al., 2013). However, when there is reduced α-secretase together with increased β-secretase activity, the amyloidogenic pathway is induced (Figure 5B). As Aβ42 increases, aggregates of Aβ form monomers that then develop into small oligomer clusters that form more stable Aβ fibrils, highly ordered crossed beta-sheet structures that align perpendicular to the fibril axis forming an extended rigid fiber (Chen et al., 2017). As more and more Aβ proteins are produced, the fibrils form a tighter and more stable structure due to the hydrophobic interactions between the amino acids side chains that stabilize into a senile plaque in the extracellular space (Chen et al., 2017). BACE-1 inhibitors (purple) target beta-secretase, reducing activity along the amyloidogenic pathway. This eventually results in the decrease in the production of the amyloid-beta (Aβ) peptides associated with Alzheimer’s disease. This figure was created with BioRender.com.

The most widely promoted hypotheses to explain the pathogenesis of AD is that neuronal damage is caused by aberrations in the processing of APP and the accumulation of Aβ due to a failure of the brain to clear Aβ (see Figure 5). Aβ was first isolated from the postmortem brains of subjects with AD and also Down’s Syndrome by Glenner and Wong in 1984, and in 1991, with the discovery of a mutation in the APP gene, (now associated with FAD) the amyloid hypothesis was proposed by Hardy and Allsop (Hardy and Allsop, 1991). Importantly, the presence of the ApoE4 allele significantly increases the risk of late-onset AD and increases the neurotoxicity of Aβ proteins (Kaplitt et al., 1996; Farrer et al., 1997).

APP is a transmembrane protein that is present in many types of cells, including neurons, and is known to play a major role in neuron synaptogenesis (Wang et al., 2009). In healthy humans without evidence of cognitive decline, Aβ is produced by the cleavage of APP by two major types of secretases: α- and β-secretase (Figure 5). When APP is cleaved by α-secretase, two fragments are generated: (i) soluble amyloid precursor protein alpha (sAPPα) and (ii) C83 (a membrane-bound C-terminal fragment) (Chow et al., 2010; Hare J, 2010). sAPPα has a role in neuroprotection and maintaining synaptic function and plasticity, which are essential for neuronal communication, learning, and memory formation (Mattson, 1997). In addition, a rat model of AD has shown that sAPPα promotes neurogenesis, which is also important for learning and memory formation (Huber et al., 1997). Most importantly, sAPPα inhibits aggregation of Aβ peptides by shifting the APP processing pathway into an anti-amyloidogenic pathway, thus preventing formation of toxic amyloid plaques generated by β-secretase. Cleavage of APP by β-secretase generates two different fragments: (i) soluble amyloid precursor protein beta (sAPPβ) and (ii) C99 (also called β-CTF), a membrane-bound fragment that is further processed by γ-secretase (Qiang et al., 2017) to yield several different beta-amyloid peptides, of which Aβ42 is the most common, and the basis of the amyloidogenic pathway (de Paula et al., 2009). γ-secretase is a protein complex made of many subunits including PSEN1, PSEN2, nicastrin, anterior pharynx-defective 1 (APH-1), and presenilin enhancer 2 (PEN-2) (Zhang et al., 2014). As mentioned previously, although a mutation in the PSEN1 gene is more commonly associated with earlier onset of FAD, a mutation in PSEN2 can also contribute. In fact, both PSEN1 and PSEN2 play important roles in forming γ-secretase’s catalytic subunit that if mutated, leads to alternations in the APP cleavage process and results in increased production of longer and more aggregation-prone forms of Aβ (Kabir et al., 2020).

Co-morbidities, such as insulin resistance in patients with T2D, enhance the buildup of Aβ plaques and advanced glycation end-products (AGEs) can modify Aβ peptides and accelerate aggregation of soluble Aβ peptides (Vitek et al., 1994). There is a strong link between insulin deficiency, insulin resistance, diabetes, and AD, with some research referring to AD as ‘type 3 diabetes’ (Steen et al., 2005; de La Monte and Ward, 2008). In addition, patients who are subjected to elevated oxidative stress, or inflammation, such as during frequent infection (also see section 3.6), are prone to developing abnormal Aβ plaques (Sharma and Kim, 2023), and Aβ42 itself has pro-oxidant activity (Ganguly et al., 2017; Butterfield and Boyd-Kimball, 2018). As previously discussed, studies have shown that Aβ42 levels decrease in CSF but increase in plasma (Teunissen et al., 2018), implying the measuring of plasma Aβ42 to monitor AD progression; however, the data has questioned its diagnostic utility.

Normally, Aβ is rapidly cleared by microglia, which act as the phagocytic cells in the CNS (Liu et al., 2021). However, in elderly people, the ability of microglia to phagocytose and degrade Aβ is decreased, resulting in decreased clearance and accumulation of Aβ inside neurons, eventually triggering inflammation, reduced neuronal communication, tau tangles, and ultimately death and degeneration (Lee and Landreth, 2010; Mucke and Selkoe, 2012; Bloom, 2014).

The argument that the buildup of amyloid plaques is the cause of AD has been vigorously disputed (Kepp, 2017), and this skepticism is supported by disappointments in the results of clinical trials with anti-Aβ MABs (Reiss et al., 2021). Skepticism supports the need to explore outside of the amyloid hypothesis for alternative targets (Karran and Hardy, 2014; Tse and Herrup, 2017; Herrup, 2022; Kaur et al., 2024). Amyloid plaques may start 20–30 years prior to evidence of cognitive function thereby raising issues over early detection, when to initiate treatment, and the potential of significant side-effects arising from long-term chronic treatment with drugs (Jansen et al., 2015). In addition, a significant percentage of patients with dementia are amyloid negative (Beach et al., 2012; Serrano-Pozo et al., 2014); in contrast, there are reports that elderly people with normal cognitive function have elevated levels of Aβ plaques (Aizenstein et al., 2008; Kepp, 2017)

β-secretase (BACE-1) inhibitors have been developed to decrease the level of Aβ proteins. However, Phase 2/3 clinical trials of subjects with mild-moderate AD with atabecestat and verubecestat were stopped due to low clinical efficacy with cognitive decline greater than in the placebo group (Egan et al., 2019; Henley et al., 2019). Similarly, lanabecestat also failed to slow cognitive decline and raised concerns over psychiatric adverse events (Wessels et al., 2020). Conceivably the failure of BACE1 inhibitors is linked to β-secretase targeting not only APP but other proteins, thus contributing to toxic side effects (Bazzari and Bazzari, 2022). It is argued that targeting γ-secretase with specific modulators (GSMs) will provide better specificity (Hur, 2022).

#### 3.3.1 Anti-amyloid (anti-Aβ) monoclonal antibodies (MABs)

AChEIs and NMDARIs provide only symptomatic relief to patients with AD whereas the argument for targeting amyloid Aβ aggregates in the brain is that this will terminate the downstream pathophysiological sequalae and potentially reverse the disease process. Passive immunization of APP transgenic mice with the MAB, mAb158, which is highly selective for protofibrils, showed improvements in learning and memory, although there was a minimal effect on amyloid burden (Lord A et al., 2009). These data suggest that soluble amyloid protein oligomers, rather than insoluble amyloid plaques, induce neural toxicity in AD patients and MABs that selectively target soluble oligomers should have better outcomes in patients with AD (Lord et al., 2009; Tolar et al., 2020). Several anti-Aβ MABs have been developed and tested and include first generation bapineuzumab, solanezumab, and crenezumab, the latter being highly homologous to solanezumab, and second generation aducanumab, lecanemab, gantenerumab, and donanemab. Table 1 provides a summary of the six MABs that have entered clinical trials. Only two appear to offer therapeutic benefits, albeit with considerable controversy, and only one, as of April 2024 has been approved–namely, lecanemab. The following discussion will focus on lecanemab, donanemab (currently under review), and aducanumab (provisionally approved but withdrawn from the market in early 2024). Table 2 includes a comprehensive summary of drug targets including additional information on aducanumab, lecanemab, donanemab

**TABLE 1:** Monoclonal Antibodies (MABs) Developed to Target Amyloid Plaques. Abbreviations: ARIA-E − Amyloid-Related Imaging Abnormalities with Edema; ARIA-H microhaemorrhages, or small hemorrhages and hemosiderosis; Clinical Dementia Rating Sum of Boxes (CDR-SB), is a scale that assesses both function and cognition; Institute for Clinical and Economical Review–ICER; IV–intravenous administration; SC–subcutaneous administration.

MAB	Status	Results	Negative effects	Controversies	Key references
Aducanumab	2 trials of patients with MCI and confirmed amyloid pathology aged 50–85: 1638 subjects in EMERGE and 647 in ENGAGE.	Primary objective met in EMERGE but not in ENGAGE–based on Clinical Dementia Rating Sum of Boxes (CDR-SB)	i. ARIAs, were experienced by more than 40% of patients taking aducanumab and dose-dependent, of whom 7.5% were symptomatic (Sevigny et al., 2016). ARIASs were more frequent in patients carrying the ApoE4 allele, and most frequent for those who carry the ApoE4 4/4 *versus* 4/3 or 3/3 alleles (Kwan et al., 2020)	i. Three FDA resigned alleging absence of evidence of effectiveness	Mahase 2021a, b, c
IV (human MAB derived from a blood lymphocyte library of elderly people without any evidence of cognitive impairment)Biogen	FDA approved June 2021		ii. Conflicting evidence from 2 trials presented at the November 2020 meeting of the FDA’s Peripheral and CNS Drug Advisory Committee meeting with some data favouring the placebo (Dr. Krudys)	ii, 2021 not approved in Europe	Budd Haeberlein et al. (2022)
Has strong avidity for epitope-rich insoluble amyloid plaques and oligomers rather than amyloid monomers. spares amyloid monomers, which have putative protective actions half-life of approximately 25 days (Beshir et al., 2022)	July 2021 use restricted to MCI.			iii. June 2022 Biogen withdraws review from Health Canada	Arndt et al. (2018)
	In early 2024 Biomega announces availability of aducanumab will end in late 2024				Tolar et al. (2020)
					Decourt et al., 2021; Haddad et al., 2022
					Whitehouse and Saini (2022)
					Rahman et al. (2023)
					Wojtunik-Kulesza et al. (2023)
					Ackley et al., 2021
					Richard et al. (2022)
Bapineuzumab (humanized MAB)	2012: Failed two Phase III trials	Failed to produce significant cognitive improvements; despite lowering Aβ, and also phosphorylate tau in CSF.	First MAB to be associated with ARIA-E		Miles et al. (2013)
Pfizer and Johnson and Johnson	Discontinued 2013		6% developed aseptic meningitis		Abushouk et al., 2017
SC					
Binds to the N-terminal of Aβ residues 1–5					
Donanemab (humanized)	TRAILBLAZER-ALZ Phase III trials	Significant improvement noted at 76 weeks			Mintun et al., 2021
Eli-Lilly					Sims et al. (2023)
IV					
Selectively targets amyloid plaques. See also Table 2					
Gantenerumab (human)	2017 Failed in Phase III	Reduced Aβ plaques but did not slow cognitive decline at 116 weeks			Ostrowitzki et al., 2017
Hoffman-La-Roche	2023 GRADUATE I and II trials				Bateman et al. (2023)
SC					
Targets insoluble plaques					
Lecanemab (human)	approved by the FDA in July 2023	CLARITY AD trial with 1795 participants for 18 months. Moderately less decline based on CDR-SB scale	ARIA-E and	April 2023 ICER report raised concerns about cost-effectiveness of lecanemab for AD.	Van Dyck et al. (2023)
IV			ARIA-H and concerns over neuroinflammation and brain shrinkage, edema and some deaths	Longer trials also recommended	Couzin-Frankel & Piller (2022)
Biogen and Eisai			ARIA higher in ApoE4 patients		Piller (2022)
Targets both oligomers and plaques					Couzin-Frankel, (2023)
See also Table 2					
Solanezumab (humanized from mouse)	Phase III EXPEDITION 1, EXPEDITION 2. showed + ve results, but failed in EXPEDITION 3. Also failed in The Anti-Amyloid Treatment in Asymptomatic Alzheimer’s (A4) study	After 240 weeks there was no slowing of cognitive decline in preclinical Alzheimer’s disease	ARIA-E >1% in each group. ARIA-H in ∼30% and similar in placebo group	Hoffman La Roche withdraws support in 2019 for continuation of trials with crenezumab	Sperling et al. (2023)
Eli-Lilly		Similarly, the 2014 phase II studies BLAZE and ABBY failed to show significant improvement with crenezumab	In 2022 NIH stated that crenezumab failed for treatment of early onset AD.		Cummings et al. (2018)
High affinity (picomolar) binding to monomeric aβ (amino acid sequence KLVFFAED)					
Highly homologous to crenezumab					
Genentech					
IV					

**TABLE 2:** Summary of drugs and drug targets for the treatment of Alzheimer’s disease.

Drug class	Drugs	Status and use	Properties and common side effects	Key references and notes
AChE inhibitors (AChEIs)	Tacrine (CognexR), donepezil (AriceptR), rivastigmine (ExelonR), galantamine (ReminylR)	Used for both mild to moderate AD.	All AChEIs inhibit both AChE and butyrl (pseudo) BuChE but with different affinities	Tacrine
		Tacrine approved in 1993 but withdrawn in 2021 due to liver toxicity; donepezil, approved in 1996	Tacrine is a non-specific AChEI; donepezil has ∼1000 selectivity for AChE and also has a long plasma half-life (60–90 h) that facilitates daily dosing and also close to 100% bioavailability; rivastigmine, is a non-specific AChEI, which in addition to oral formulations is also available as a transdermal patch; galantamine in addition to AChEI actions is also a positive allosteric inhibitor of nicotinic receptors	Gracon et al., 1998; Jarrott, 2017; Watkins et al., 1994; Blackard et al., 1998; Samuels and Davis, 1997
		Meta-analysis by Birks and Harvey, 2018, concluded that donepezil use resulted in better scores on ADL (activity of daily living)	The common side-effects are similar for all AChEIs and mainly	Donepezil
		Donepezil is widely used and in combination with the NMDA receptor modulator, memantine for symptomatic relief, and in in 2001 for severe AD.	GI-related, but reportedly less for donepezil–see Ali et al., 2015	Atri, 2019; Birks and Harvey, 2018; Marucci et al., 2021; Fish 2011
		The third AChEI to be approved was rivastigmine in 1997, and the fourth, galantamine, was approved in 2001		Sugimoto et al. (2000)
				Rivastigmine
				Birks and Harvey, 2018
				Marucci et al., 2021
				Multum, 2019
				Galantamine
				Wang et al., 2007
				Mohammad et al., 2017; Brodaty et al., 2005; Kavanagh et al., 2011; Jiang et al., 2015
NMDA Receptor Antagonist	Memantine (AxuraR, EbixaR, NamendaR)	A low-affinity uncompetitive antagonist of NMDAR. Approved by FDA in 2003 for symptomatic relief of mild to moderate AD and based on the results of two clinical trials (Kavirajan, 2009) Memantine is available in oral formulations including extended-release (ER). Memantine has high bioavailability (approaching100%) and a plasma half-life of 60–70 h. Frequently used in combination with donepezil	Well-tolerated with headache, blurred vision, dizziness as infrequent side effects (Kuns et al., 2024)	(I). Affinity for NMDAR *versus* other receptors
			Memantine is safe with co-morbidities (diabetes, and co-administration with metformin or glyburide); co-use with AChEIs is safe	Chen and Lipton, 2005; Johnson and Kotermanski, 2006; Seeman et al., 2008. (ii). Supportive clinical trial data: Kavirajan et al., 2009; Periclou et al., 2004; Noetzli and Eap, 2013
			Based on meta-analysis (Blanco-Silveste et al. (2018) discontinuation rates for memantine are higher than for placebo	Matsunaga et al., 2015
				Kishi et al., 2017. (iii) Cochrane Report: McShane et al., 2019
				(iii) Use with co-morbidities
				Freudenthaler et al. (1998), Periclou et al. (2004), Shua-Haim et al. (2008)
Combination therapy AChEI plus NMDAR antagonist (donepezil + memantine)	Donepezil + memantine. (NamzaricR)	Fixed dose combinations approved in 2014 for patients with moderate to severe AD.	Overall, the evidence suggests that side-effects of combination therapy is more effective than monotherapy and that side effects are no greater than for monotherapy treatment with either AChEI or memantine (Kuns et al., 2024)	(i). Positive data based on ADCS-ASL scores (Tariot et al., 2004; Grossberg et al., 2013)
			Meta-analysis suggests combination many be more effective for non-AD dementias	(ii). Contradictory data: Atri et al., 2008; Porseinsson et al., 2008; Howard et al., 2012; Chen et al., 2017a)
				Also see
				Deardorff and Grossberg, 2016; Calhoun et al., 2018; Saint-Laurent Thibault et al., 2015
				(iii). Meta-analysis for combination therapy AD *versus* other dementias
				Blanco-Silvente et al., 2018; Knight et al., 2018; Veroniki et al., 2022
MABs directed at amyloid (Aβ) proteins	Bapineuzumab, solanezumab	First generation chimeric humanized zumabs failed clinical trials	Infusion reactions are the most common SEs with an incidence of ∼25%	(i). Based on Aβ accumulation in post-mortem brains
(Anti- Aβ MABs)	crenezumab	Aducanumab (a numab–fully human MAB) approved with considerable controversy in 2021, but Biomega announced availability will end in late 2024	Reports of cerebral edema in addition to cost of drug and associated costs of CSF and MRI monitoring for ARIAs may limit use and wider global use	Glenner and Wong, 1984; Hardy and Allsop, 1991; Hardy and Higgins, 1992
	aducanumab (AduhelmR)	Lecanemab approved in 2023 and based on positive data from CLARITY-AD trial. Based on TRAILBLAZER-ALZ 2 RCT approval of docanemab anticipated in late 2024	Insufficient data to know long-term therapeutic efficacy and effects of anti-Aβ MABs	Also see: Spirling et al., 2011
	lecanemab (LeqembiR)		Considerable controversy over the approval and effectiveness of anti-Aβ MABs	Mahase 2021a, b, c
	gantenerumab			Rahman et al., 2023
	docanemab (LY3002813, or N3pG)			Wojtnik-Kulesza et al. (2023)
				Controversies
				Teich and Arancio 2012; Ackley et al., 2021; Reiss et al., 2021; Richards et al., 2021; Herrup, 2022; Whitehouse and Saini, 2022; Kaur et al., 2024
				Additional and longer clinical trials with lecanemab are ongoing (van Dyck et al., 2023) - results should help clarify how beneficial this class of drugs are re. long-term treatment of AD and whether anti- Aβ MABs slow cognitive decline
β-secretase (BACE1) inhibitors)	Atabecestat; verubecestat	Failed Phase 2/3 clinical trials	Low clinical efficacy with cognitive decline greater than with placebo and concerns over psychiatric side effects. High incidence of side effects linked to ‘on target’ effects of β-secretase on proteins other than APP.	Henley et al., 2019
	Lanabecestat			Egan et al., 2019; Wessels et al., 2020
	Targets APP (amyloid precursor protein)			See also McDade et al., 2021
γ-secretase	Selective modulators (GSMs) of γ-secretase predicted to have fewer side effects than BACE1 inhibitors	None tested		Hur (2022)
Tau-inhibitors	Semoriinemab, tilavonemab, gosuranemab target the N-terminal region of tau	Failed to show significant benefits as in the TANGO trial with the humanized MAB, gosuranemab	Insufficient data to determine significance of side effects in humans; studies in mice did not show significant issues	Hoskin t al., 2019; Shulman et al., 2023; Sperling, 2023
	RNA-bases anti-sense oligonucleotide targeting tau (IONIS in partnership with Roche). Phase 1b study in progress (NCT03186989)	Data suggests that more specific targets are needed and/or combined therapy with multiple targets		Teng et al., 2022
				Panza and Lozupone (2022)
				September 2023 IONIS entered into an agreement with Roche for the further development of antisense RNA therapies for the treatment of AD and Huntington’s Disease (https://ir.ionispharma.com/news-releases/news-release-details/ionis-enters-agreement-roche-two-novel-rna-targeted-programs)
NSAIDs	Numerous NSAIDs including aspirin, naproxen, ibuprofen, and coxibs (celecoxib)	Early positive data based on retrospective studies not supported by later meta-analysis and Cochrane Review in 2012 that concluded there was no evidence to support either the use of aspirin, NSAIDs, selective COX-2 inhibitors (coxibs), or steroids for the prevention or treatment of AD.	Chronic use of NSAIDs linked to risk of increase in GI and cardiovascular morbidity and mortality, and elevated cardiovascular risk for coxibs–Schjerning et al., 2020	(i). Supportive: McGeer et al., 1990, 1996; Szekely et al., 2004
		The results of the 2019 clinical trial, INTREPAD, with the NSAID, naproxen were negative		In t’Veld et al., 2001; Pasinetti 2002
				(ii). Cochrane Review Jaturapatporn et al., 2012
				(ii). INTREPAD data (with naproxen)–no benefits in AD (Meyer et al., 2019)
				(iii). NSAIDs and coxib use and elevated risk in elderly patients (Wehling, 2014)
Anti-diabetes drugs	Metformin and GLP-1 receptor agonists	Support provided by pre-clinical and retrospective clinical data as well genetic analysis. Diabetes increases the risk of AD and benefits of drugs may be secondary to improving metabolic control in patients	Side effects with metformin are primarily GI.	El-Mir et al., 2008
		Metformin in Alzheimer’s–a Phase II/III trial (NCT04098666) with long-acting metformin in non-diabetes subjects with early and late MCI. Results expected in late 2026	GLP-1 receptor agonists frequently cause nausea, vomiting, loss of appetite	Campbell et al., 2018
				Zheng et al., 2022
				Nogaard et al., 2022
				Nowell, et al. (2023)
ApoE and statins	Gene therapy to target ApoE4 homozygotes	Lexeo Therapeutics, Phase I/II Clinical Trial, NCT03634007, on-going	Data on gene therapy clinical trial expected in late 2024	Controversy re statins are beneficial in AD Wagstaff et al., 2003; Zandi et al., 2005
	See Rosenberg et al., 2018	Using an adeno-associated virus gene transfer vector that expresses the cDNA coding for human APOE2		Statins may reduce risk of AD, cognitive decline and mortality Kostapanos and Elisaf, 2017; Jeong et al., 2021; Nowak et al., 2022; Olmastroni et al., 2022; Murphy et al., 2023
				Targeting APOE4–see Hunsberger et al., 2019; Vecchio et al., 2022
				Expression of ApoE4 damages pericyte function and integrity of BBB
				Armulik et al., 2010; Nishitsuji et al., 2011; Halliday et al., 2016; Zhou et al., 2022)
Viruses and Vaccinations	Several viruses have been linked to increasing the risk of AD including Herpes Simplex (HSV-1), SARS-CoV-2	Viral infections linked to increase in neuroinflammation, and increases in β- and γ-secretase activities and enhancing APP processing and tau kinases	Vaccinations have been shown to reduce the risk of AD (Wu et al., 2022)	Wozniak et al., 2007
			A prospective study (n = 49), with the *Bacillus* Calmette–Guérin (BCG) vaccine for tuberculosis has provided positive data that vaccines against AD (Dow et al., 2022)	De Vlieger et al., 2022
				Kitazawa et al. (2011)
				Patients with “Long COVID” experience sleep disruption, fatigue, anxiety, depression and what has been referred to as “brain-fog” (inability to focus, loss of memory, and difficulty to conduct normal activities) that can persist for months (Venkataramani and Winkler, 2022). Whether protection is provided by COVID-19 vaccinations remains to be analysed

The failure of several clinical trials, deaths of a number of subjects in the trials, controversy over the approval process, and the cost of the anti-Aβ MABs has added to earlier skepticism that these MABs will proce to be “game-changers” in the treatment of AD (see Kaur et al., 2024; Table 2). Furthermore, PET analysis suggests as many as 25% of patients diagnosed with mild to moderate AD are amyloid negative AD (Beach et al., 2012; Serrano-Pozo et al., 2014; Herrup, 2015). In addition, racial differences, such as reports of significantly lower CSF levels of the biomarkers T-tau and p-tau18 in African-Americans diagnosed with AD (Morris et al., 2019), complicate the assessment of the therapeutic efficacy of treatment and require careful design of clinical trials to ensure appropriate ethnic representation. Another concern is evidence that Aβ may play an important role in water homeostasis in the brain via association with the aquaporin AQP4 water channel, expressed primarily in the CSF and brain interface, which is upregulated in the brains of patients with AD (Moftakhar et al., 2010; Lehrer and Rheinstein, 2023; Maroli, 2023). This putative link between Aβ and the regulation of water homeostasis could explain the frequent occurrence of amyloid-related imaging abnormalities (ARIA) in patients treated with MABs.

Two types of ARIA are seen with the use of anti- Aβ MABs. ARIA-E is a vasogenic edema, whereas ARIA-H involves microhemorrhages and hemosiderosis (Barakos et al., 2022). ARIA-H is usually preceded by cerebral amyloid angiopathy as the amyloid proteins weaken the blood vessel endothelium and increase the risk of bleeding (Goos et al., 2010). Both forms of ARIA are usually transient and minimally symptomatic; however, when symptomatic, ARIA presents with headaches, dizziness, confusion, visual disturbance, nausea, seizures, and even death, as has been associated with the use of the recently approved MAB for AD, lecanemab (Piller, 2022; Withington and Turner, 2022). It is conceivable that a refinement in the epitope recognized by the MAB may reduce the incidence of ARIAs and enhance plaque removal. In a mouse model of AD targeting Aβ(p3-42) promotes plaque removal, whereas targeting Aβ(p1-42) was associated with a higher number of microhemorrhages (Demattos et al., 2012).

Abucanumab was provided accelerated approval by the FDA in June 2021 and the first new drug approved for AD since 2003 (Rahman et al., 2023). Approval was based on two Phase III trials, EMERGE and ENGAGE, wherein a total of 1,643 and 1,647 subjects with MCI or mild AD were enrolled to take either low-dose or high-dose aducanumab once every 4 weeks for a period of 18 months; lower doses were given to subjects carrying the ApoE4 allele due the higher risk of ARIA. In July 2021 the FDA restricted use to patients with MCI (see Table 1; Mahase, 2021a; b). In part, the controversy relates to the differences in the data from the EMERGE *versus* ENGAGE trials. Data from the EMERGE trial showed that aducanumab improved several cognitive and functional assessment scores (Cummings et al., 2021). However, data from the ENGAGE trial showed no benefit and, of note, it was the data from the higher dose and recruitment of additional patients in the EMERGE trial that provided the positive benefit (Knopman et al., 2021; Budd Haeberlein et al., 2022). A meta-analysis reported that based on the PET data aducanumab treatment lowered amyloid load and increased p181-tau in the CSF. However, although statistically significantly the improvement in the ADAS-Cog was small and no change was noted in MMSE (Avgerinos et al., 2021).

Whitehouse and Saini (2022) argued that based on questionable efficacy and the risk of significant side effects, the approval of abucanumab should be withdrawn. Furthermore, neither the European Medicines Regulatory Network nor the UK regulatory agency granted approval for aducanumab (Mahase, 2021c). In early 2024, Biogen announced that it would stop developing and marketing aducanumab (Alzheimer’s, 2024) and contributing factors likely included the decision of the US Centers for Medicare and Medicaid Services (CMS) to restrict reimbursement to participants in a CMS-approved clinical trial (Cummings, 2023; Wojtunik-Kulesza, et al., 2023). Additional factors that also apply to similar anti-Aβ MABs include the estimated annual $28,000 cost of the drug, and the extra costs needed for monthly CSF analysis and MRI reports, the later required for monitoring signs of ARIAs–potentially the most serious side effects of anti-Aβ MABs (Sperling et al., 2011; Wojtunik-Kulesza, et al., 2023).

Lecanemab was granted accelerated approval by the FDA on January 2023 and based on data from the CLARITY AD Phase III trial, full approval was granted in July 2023 for AD subjects with MCI. Lecanemab, like aducanumab, is a humanized IgG1 monoclonal antibody that selectively binds to soluble amyloid protofibrils and initiates the clearance of both protofibrils and amyloid plaques (McDade et al., 2022). CLARITY AD was an 18-month trial that involved 1,795 patients with MCI or mild dementia who received weekly IV infusion of 10 mg/kg of lecanemab; all the participants had confirmed pre-trial evidence of amyloid deposits observed by PET scan. End of trial data demonstrated, compared to placebo, a significant reduction in amyloid plaque burden, a 27% slowing of cognitive decline measured by CDR-SOB, 37% slowing of the decline of ADCS-MCI, and based on ADAS-Cog measurements, a 26% reduction in the decline of cognition (Van Dyck et al., 2023). A news release from Biogen and Eisai on the 29 November 2022 reported that subjects treated with lecanemab had reduced pTau181 and T-tau in the CSF together with a reduction in tau pathology, and lower levels of Glial Fibrillary Acidic Protein (GFAP) (a marker for astrocyte activation during neuroinflammation) and neurogranin were observed in the plasma (Biogen, 2022). Data from longer trials are required to determine how beneficial anti-Aβ MABs are for the long-term treatment of AD and whether they slow cognitive decline (van Dyck et al., 2023).

Although the data from the CLARITY-AD trial have been interpreted as positive it is important to note that the side effects associated with lecanemab were comparable to those attributed to aducanumab. The most common side effects were infusion-related reactions with approximately 40% of patients requiring acetaminophen or antihistamine prior to infusions. Subjects receiving lacenemab experienced more ARIA-E or ARIA-H (17%) compared to the placebo group (9%), more commonly in those carrying the ApoE4 gene allele, particularly homozygotes. Importantly, AD patients who were receiving thrombolytics were at higher risk of ARIA-H. Other concerns, as expressed by the European Alzheimer’s Disease Consortium Executive Committee relate to the cost of the drug estimated at $26,500/year, and access currently limited to those with MCI but not those with moderate to severe AD (Jönsson et al., 2023). Kwan et al. (2020) raised the issue that data was needed patients who were Aβ positive but asymptomatic for AD. Additional concerns have been expressed regarding the trial’s racial demographics, with approximately 77% of participants white, followed by 17% Asian and only 2.6% Black (and among participants from the U.S., 94.5% white with the rest either Asian or Black) (Van Dyck et al., 2023). Concerns over the race/ethnicity makeup have been raised for clinical trials with other drugs (Turner et al., 2022). Collectively, these concerns add to the questions raised in the Anti-Amyloid Treatment in Asymptomatic Alzheimer’s (A4) study (Stopping AD before Symptoms Begin) as to screening for AD and when treatment should begin (Sperling et al., 2014), the results of which were not positive for the MAB, solanezumab, despite following patients for 240 weeks (Sperling et al., 2023). Results of the TRAILBLAZER-ALZ 2 RCT with donanemab were released in July 2023, with comparable positive outcomes to lecanemab, albeit also with limited clinical benefits, similar limitations, and questions over whether these MABs halt the progression of AD or improve quality of life for those receiving the drugs (Sims et al., 2022; O’Leary, 2023).

In conclusion, as of early 2024, it is clearly premature to predict whether the anti-Aβ MABs will prove to be a provide the “magic bullet” for the treatment of AD (Kaur et al., 2024). As stated in a July 2023 editorial in JAMA the beneficial effects of both lecanemab and donanemab are modest, and ongoing research is crucial to fully understand the clinical effectiveness and long-term safety profile, particularly concerning potential adverse effects. Collectively, although the data show that anti-Aβ MABs do slow the rate of functional and cognitive decline in some patients, the results also add to the evidence that amyloid is not the only factor responsible for the progression of AD (Kepp, 2017; Widera et al., 2023).

### 3.4 Tau hypothesis

**FIGURE 6:**
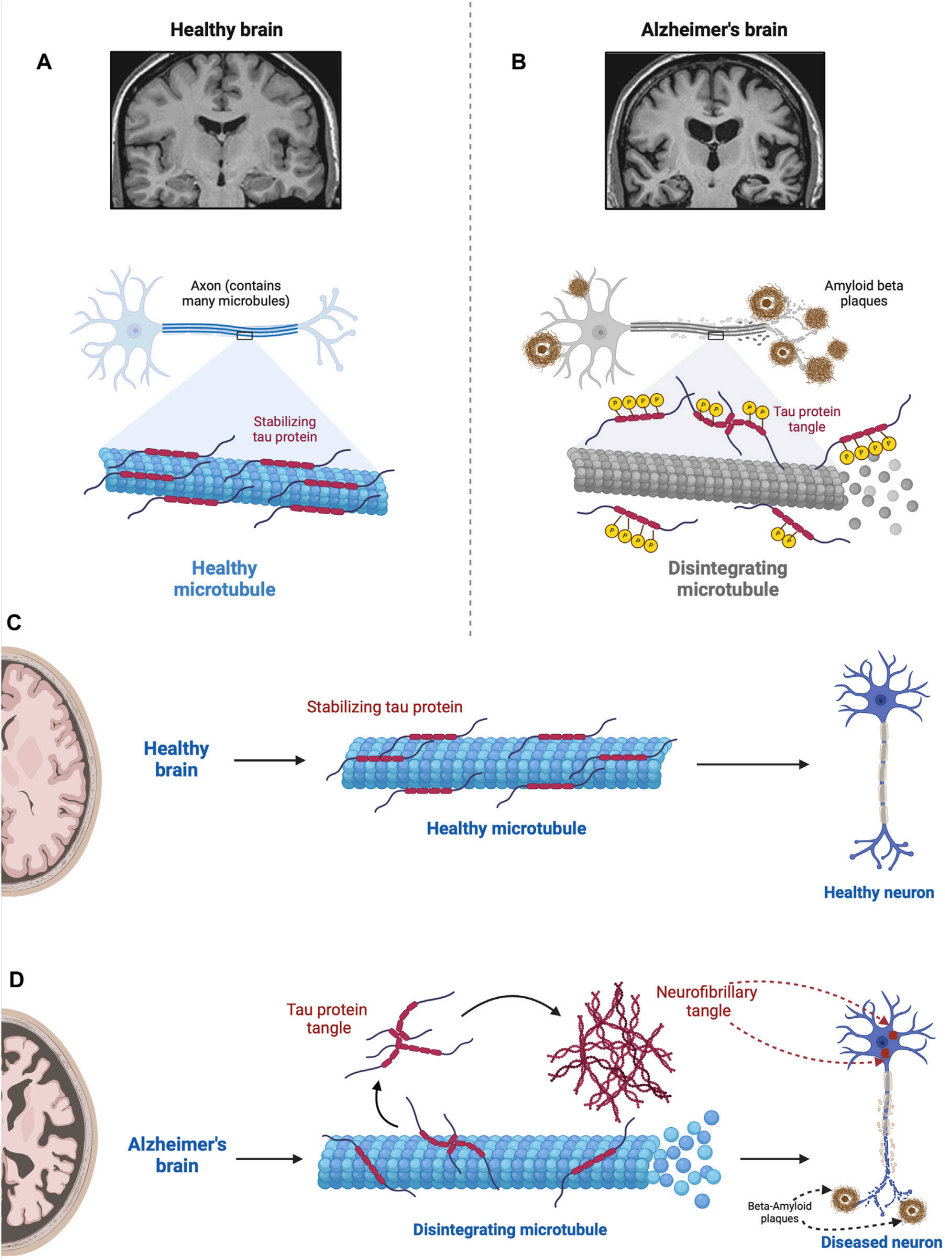
Impact of hyperphosphorylated tau on Microtubule Stability. This figure illustrates the detrimental effect of hyperphosphorylated tau proteins on microtubules. In the normal state **(A)**, tau stabilizes microtubules via a balance between negatively and positively charged residues in their monomers (Mukrasch et al., 2007; Jho et al., 2010). In the absence of any pathological conditions, the balance between these positively and negatively charged molecules keeps tau proteins attached to microtubules where they assist in essential molecular transport. However, when tau becomes hyperphosphorylated in the diseased state **(B)**, via the src family tyrosine kinase, fyn (Lee et al., 2004), tau proteins become less and less positive weakening the electrostatic force between tau and microtubules (Fischer et al., 2009). This results in microtubule disintegration impairing ubiquitin proteasome-mediated autophagic clearance of Aβ (Cowan et al., 2010; Weng and He, 2021). In addition, tau neurofibrillary tangles within the neuron physically obstruct the movement of cellular components along the microtubules; the density of neurofibrillary tangles and severity of the pathology correlate with the level of cognitive impairment (Cowan et al., 2010; Nelson et al., 2012). In the normal state **(A)**, tau stabilizes microtubules, which are essential for cellular structure and transport. However, when tau becomes hyperphosphorylated in the diseased state **(B)**, it loses its stabilizing ability, resulting in microtubule disintegration. This disruption compromises cellular structure and function, contributing to the pathogenesis of Alzheimer’s disease. Figures C and D compare the role of tau in the normal brain **(C)** with the AD brain **(D)**. D shows microtubule disintegration, neurofibrillary tangle formation, and amyloid plaque deposition and accumulation. This figure was created with BioRender.com.

As summarized in Figure 6 the tau hypothesis is based on observations that AD is associated not only with an accumulation of Aβ but also aggregates of misfolded tau protein (see Lee et al., 2001; Frost et al., 2009). In AD, aggregates of Aβ are implicated in causing hyperphosphorylation of tau proteins (Gong and Iqbal, 2008), which interact with the nuclear pore complex producing structural disruption and functional loss culminating in neurotoxicity (Eftekharzadeh et al., 2019). Tau, which in the human brain exists in six isoforms, is a microtubule-associated protein that stabilizes the microtubules that serve as the highway for transporting cellular components.

#### 3.4.1 Targeting tau

There is considerable interest in targeting tau to treat AD, but despite positive pre-clinical data, at present no approach has been approved for use in patients (Soeda and Takashima, 2020). The greater number of studies involve MABs, and the TANGO trial with the humanized MAB, gosuranemab, which like other anti-tau MABs (semorinemab and tilavonemab) directed at the N-terminal region of tau, showed no significant benefits (see Table 2; Monteiro, et al., 2023). AV-1980R/A, which targets the N-terminus of tau, has also shown promise in cynomolgus Monkeys (*Macaca fascicularis*) with a robust anti-tau antibody response, which, in theory, should reduce tau tangles (Hovakimyan et al., 2022). Subject to positive data from trials in humans this MAB could be used in patients at risk of AD.

A different approach to targeting tau is to use an antisense RNA that targets microtubule-associated protein tau and this is the focus of a randomized, double-blind, placebo-controlled clinical trial (NCT03186989) with BIIB080 (also known as IONIS -MAPTRx) (https://clinicaltrials.gov/).

### 3.5 Vascular hypothesis

The vascular hypothesis, as originally proposed in 1993 by De La Torre and Mussalvand, states that any disruption of blood supply that compromises cerebral perfusion will result in microglial activation and the build-up of neurofibrillary tangles and elevate the risk of a decline in cognitive function. This hypothesis is supported by epidemiological data as well as ultrastructural data from postmortem brains of subjects with AD that show extensive pathological changes in cerebral capillaries.

**FIGURE 7:**
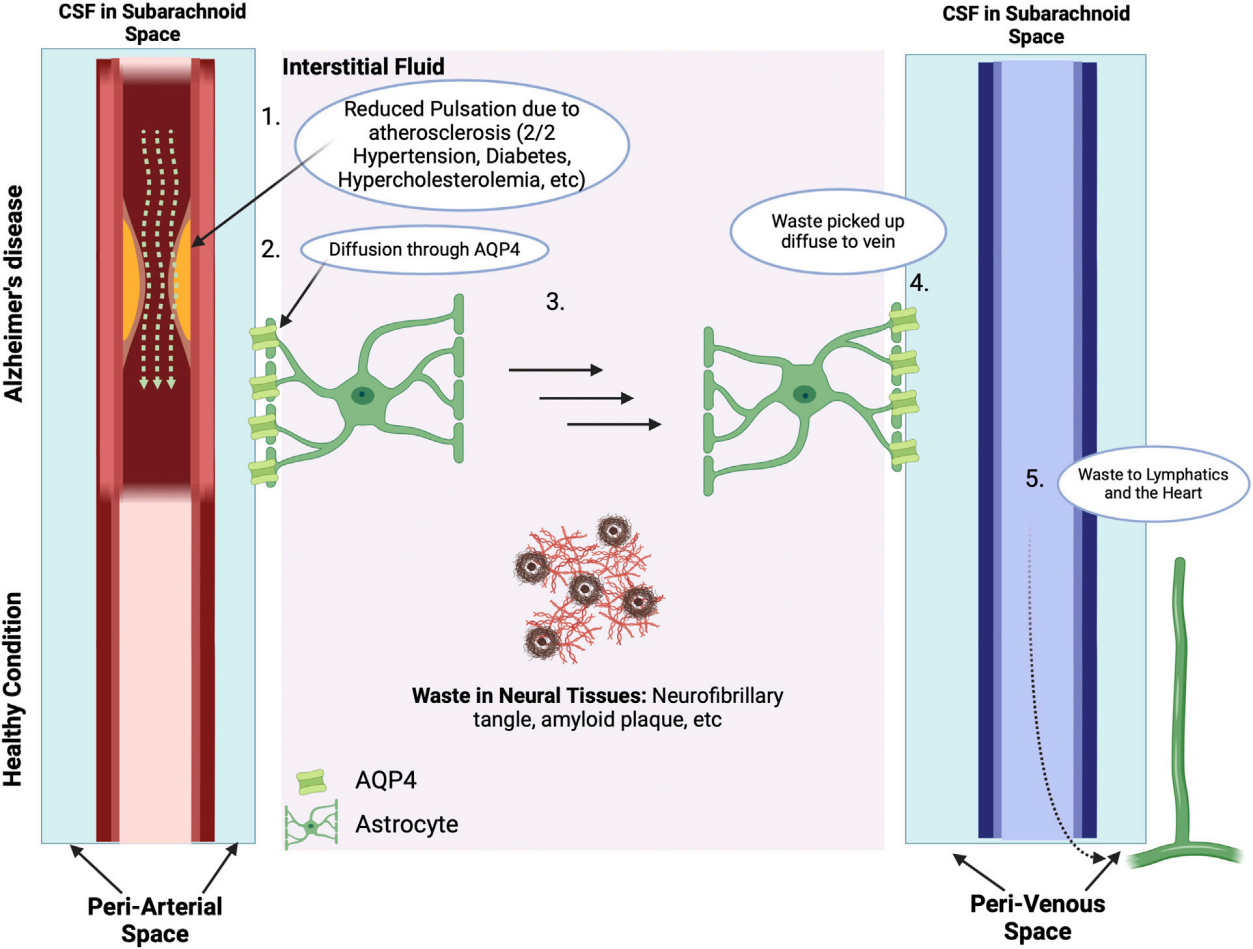
The Vascular Hypothesis. The Blood-Brain Barrier and ApoE4: There are many waste products in the interstitial space, including Aβ and tau proteins and neurofibril tangles. With normal cerebral vascular function and pulsatile blood flow, the direction of CSF flow is from the periarterial space to the interstitial space to perivenous space. While the interstitial fluid crosses the AQP4 channel on the astrocytes of the perivenous space, the waste products in the interstitial space get carried along to the perivalvular space and to the lymphatic system. However, for subjects with vascular abnormalities, such as due to atherosclerosis, the decreased pulsatile blood flow drives CSF flow from the periarterial to interstitial to perivenous space, thus resulting in accumulation of beta amyloid and tau protein in the interstitial space. The presence of amyloid plaques in turn causes additional vascular dysfunction by damaging nearby blood vessels and disrupting the regulation of blood flow in the brain (Thomas et al., 1996). Subjects who carry the ApoE4 genotype have a reduced ability to clear Aβ, which then accumulates in brain microvessels and parenchyma (Martel et al., 1997), and is then associated with reduced cerebral blood flow and metabolism across multiple cortical regions, increasing the risk of hypoxic brain injury (Small et al., 1995; Mielke et al., 1998; Kim et al., 2013). In addition, cognitively normal ApoE4 carriers show significant age-related deficits in cerebral perfusion as they age, which increases the risk of AD (Thambisetty et al., 2010; Liu et al., 2013). Carrying the ApoE4 also heightens the risk of pericyte dysfunction thereby reducing the critical role of pericytes in maintaining the integrity of the blood brain barrier (BBB) thereby allowing Aβ and other inflammatory molecules to penetrate the CNS, inducing neuroinflammation and accelerating AD in part via inhibition of the anti-inflammatory effects of the TREM2-DAP12 complex on microglia (Armulik et al., 2010; Nishitsuji et al., 2011; Halliday et al., 2016; Fitz et al., 2021; Iannucci et al., 2021; Zhou et al., 2023). Data from ApoE4 carriers shows that the breakdown in the BBB starts in the medial temporal lobe, which is the part of the brain critical for cognitive function (Montagne et al., 2020). In consequence, inflammation and oxidative stress associated with both vascular dysfunction and Aβ worsen the damage caused by each factor, creating a feedback loop that accelerates AD progression. This figure was created with BioRender.com.

Cerebral hypoperfusion not only results in hypoxia and reduced nutrient delivery to the brain but also hinders the adequate clearance of metabolic waste products, including Aβ proteins. The primary route for Aβ clearance from the brain is via the glymphatic system - a combination of astrocyte (a type of glial cell) and lymphatic system that serves as a perivascular transit network linking the CSF and interstitial solutes that serves as the brain’s clearance system (Iliff et al., 2012). As illustrated in Figure 7, CSF produced in the choroid plexus passes through the subarachnoid space and crosses the periarterial space to the interstitial fluid space via aquaporin-4 (AQP4) channels present on astrocytes. Dysfunction of the glymphatic system has been associated with neurodegenerative disease (de Leon et al., 2017; Lv et al., 2021). Ablation of the meningeal glymphatic system in mice results in the accumulation of Aβ proteins (Da Mesquita et al., 2018a; 2018b).

ApoE, a glycoprotein that is instrumental in cholesterol transport, participates in lipid metabolism, and aids in the removal of Aβ proteins (Martel et al., 1997; Mahley and Rall, 2000; Marais, 2019). Humans express three ApoE genotypes, with ApoE3 being the most prevalent genotype, and ApoE2 the most protective genotype for AD, with higher risk in ApoE2/4 and ApoE3/4 heterozygotes (McCorkindale et al., 2022). Risk is also higher in females who carry ApoE4 (Riedel et al., 2016). Approximately 25% of the population express the ApoE4 genotype and it is a major contributor to LOAD (Crean et al., 2011; Di Battista et al., 2016; Montagne et al., 2020), and lowers the clearance of Aβ (Ma et al., 2018; Zhou et al., 2023), increases the transcription of AP-1, promotes tau phosphorylation (Huang et al., 2017; Barthélemy et al., 2020), and neuroinflammation (Fitz et al., 2021; Iannucci et al., 2021). Targeting ApoE4 offers another approach to the treatment of AD and a gene therapy trial directed at ApoE4 is currently being pursued (see Table 2).

A number of studies link atherosclerosis to dementia and AD; however, it is unclear whether the enhanced risk applies to all subjects with atherosclerosis. For example, a meta-analysis reported a link to carotid artery intima-media thickness (CMIT) and the risk of AD (Xie et al., 2020). Dolan et al. (2010) also concluded that the risk of AD was enhanced in those with cerebral atherosclerosis, but no significant risk was noted in those with peripheral atherosclerosis including coronary artery disease. Similarly, Dearborn et al. (2017) reported that intracranial atherosclerosis was associated with MCI and dementia, but not specifically with AD; however, the ApoE4 genotype is associated with an elevated risk of atherosclerosis and AD (Davignon, 2005). Gender also affects the risk of atherosclerosis: women are also at higher risk of developing AD and higher for those who have the ApoE4 genotype (Mielke et al., 2014; Riedel et al., 2016). Nonetheless, the between lipid levels and AD and benefits of lipid lowering drugs has been questioned (see Table 2; Reitz et al., 2004). Matsuzaki et al. (2011) demonstrated an association between hyperlipidemia and neuritic plaques seen in AD but it did not show any relationship with the levels of neurofibrillary tangles. A 2023 report of a cohort study of over 15,500 subjects with dementia and an average age of approximately 80 reported that statin use had positive effects of cognition as based on MMSE scores and favouring those taking simvastatin (Petek et al., 2023). A 2024 population-based cohort study in Hong Kong that statin use decreased the risk of dementia in patients with heart failure by ∼20% (Ren et al., 2024), and the most recent American Diabetes Association (ADA) guidelines recommend that to avoid cognitive risk in the age group 40–75, LDL levels should not exceed 70 mg/dL (1.8 mmol/L) (see also Chou et al., 2022).

Hypertension, which affects approximately 1.3 billion people worldwide, is a significant risk factor associated with AD. Pathological changes in the vasculature result in endothelial dysfunction, a reduction in vasoprotective factors including nitric oxide (NO), elevated levels of ROS, the promotion of vasoconstriction and atherosclerotic plaque formation, and a heightened risk of the formation of thrombi (Brandes, 2014). High mid-life, but not late-life, blood pressure has been correlated with AD (Gabin et al., 2017) but lower blood pressure in those older than 75 is likely a secondary phenomenon not directly linked to AD (Skoog et al., 1998). Data suggests that angiotensin receptor blockers may be the most beneficial antihypertensive drugs for patients with AD and hypertension inferring a contributing role for aberrant angiotensin-signaling in the pathogenesis of AD (Adesuyan et al., 2022, see Table 2).

There is also a racial link between expression of the ApoE4 genotype and Aβ protein levels in the brain as well as the CSF biomarkers T-tau, which are reported to be lower in African Americans who are diagnosed with AD (Morris et al., 2019). Furthermore, subjects expressing ApoE4 who also have other risk factors such as hypertension, atherosclerosis, and hypercholesterolemia, experience reduced protective benefits from anti-hypertensive drug therapy against AD (Hajjar et al., 2002; Stampfer, 2006; de Oliveira et al., 2018). Risk is further heightened by body mass index (BMI) and leptin signaling (Blautzik et al., 2018). Collectively, these findings highlight the importance of targeting modifiable risk factors as has been emphasised by several reports (see Femminella et al., 2018; Wang et al., 2018; Livingston et al., 2020; Zhang et al., 2021).

### 3.6 Virus hypothesis

As a consequence of COVID-19 and in part because therapies for other targets such as acetylcholine and glutamate excitotoxity have given ambivalent results, there has been renewed interest in the role of viral infections, and not just SARS-CoV-2, in the development of AD, and also the potential preventive benefit of vaccinations (De Vlieger et al., 2022). (SCOPUS data are presented Supplementary Figures S2, 3 for the role of viruses and vaccinations.)

**FIGURE 8:**
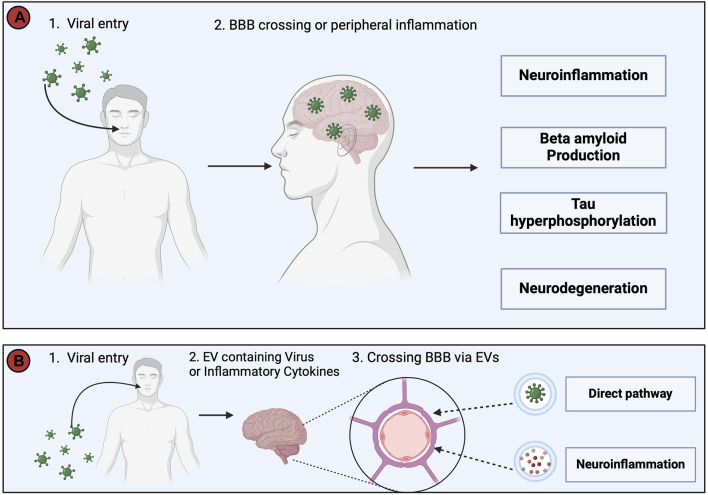
This figure depicts two potential pathways whereby viruses may trigger Alzheimer’s Disease (AD) pathology. **(A)**. The direct invasion of the virus into the Central Nervous System (CNS) or the induction of systemic inflammation by peripheral viral infection, even in the absence of direct CNS invasion. Both routes converge in the development of neuroinflammation, increased Aβ production, tau hyperphosphorylation, and neurodegeneration, collectively resulting in AD symptomatology. **(B)**. The virus utilizes extracellular vesicles (EVs) as a protective mechanism, allowing it to evade the immune system by encapsulating itself within EVs during its transit into the CNS. In addition, EVs serve as carriers for viral proteins, viral nucleic acids, and pro-inflammatory cytokines, and avoid detection by the immune system, before passing through the BBB to enter the CNS and cause infection and neuroinflammation (Yates et al., 2019; Horn and Maclean, 2021; Liu et al., 2021). The injection of EVs containing proinflammatory mediators into mice has been shown to increase activation of astrocytes and microglia, and thus supports a link between viral infections, EVs and neuroinflammation (Li et al., 2018). This figure was created with BioRender.com.

As early as 1952 links were made between the role of viral infections and AD (Sjogren et al., 1952). In 1982, it was proposed that recurring infections with human herpesvirus (HSV)-1 might be involved in the development and the progression of AD (Ball, 1982). Several other viruses including SARS-CoV-2, HIV, and spirochetal Gram-negative bacterial infections such as syphilis have been linked with the development of AD, however, a key question is: “How does a viral infection initiate or worsen AD?”. Two possible answers are summarized in Figure 8. According to the direct infection hypothesis (Figure 8A), the virus directly enters the CNS and causes neuronal death or activates an antiviral response (Seaks and Wilcock, 2020), causing neuroinflammation and AD pathology (De Vlieger et al., 2022). More frequent re-infection produces the greater cumulative damage seen in AD patients (Itzhaki et al., 2016). The indirect infection theory suggests that the virus does not necessarily need to enter the CNS but rather a peripheral viral infection induces a systemic inflammation that can cause AD pathology (De Vlieger et al., 2022). An additional mechanism (Figure 8B) involves extracellular vesicles (EVs). EVs are small sized vesicles that transport components between cells, including proteins, lipids, nucleic acids, and misfolded proteins such as Aβ and tau proteins (Hill A.F., 2019), thereby supporting the hypothesis of a prion-like transmissible process as a cause for AD proposed in 1984 (Prusiner, 1984).

Once the virus enters the CNS, an inflammatory response occurs resulting in neuroinflammation, Aβ aggregation, and formation of neurofibrillary tangles. The process becomes amplified if the microglia fail to clear the virus, enhancing the of release inflammatory cytokines and oxidative compounds (De Vlieger et al., 2022). Data from mice show that proinflammatory cytokines induce the expression of interferon-induced transmembrane protein 3 (Ifitm3) in neurons; Ifitm3 is a transmembrane molecule known to increase the activity of γ-secretase and thereby raise levels of Aβ proteins (Hur et al., 2020). The proinflammatory molecules released as a result of viral infection shift the APP processing towards the amyloidogenic pathway. Viral infection also increases the activity of Tau kinases and increases the formation of neurofibrillary tau tangles (Kitazawa et al., 2011). Viral infection is also associated with increased quinolinic acid (QA) (De Vlieger et al., 2022), produced by microglia and activated macrophages (Braidy et al., 2009). In the nematode *Caenorhabditis elegans*, QA acts as a neurotoxin and has been shown to contribute to neurodegeneration via the activation of NMDA receptors, increasing cytosolic calcium and ROS levels and ultimately depleting ATP (da Silveira et al., 2018). Collectively, these data prompt further investigations of the link between viral infections, neuroinflammation and the development of AD.

Herpes Simplex Virus type 1 (HSV-1), has been extensively studied for its role in AD. Upon initial infection, HSV-1 stays latent in the brain and in the trigeminal ganglia (Jamieson et al., 1991). During periods of acute stress or immunocompromised states, it can reactivate, resulting in viral shedding and initiating inflammation in nervous tissue leading to the release of ROS from microglial cells. HSV-1 infection itself may shift APP processing into the amyloidogenic pathway by increasing the expression of β-secretase and nicastrin, a component of γ-secretase, thus increasing the production of amyloid proteins (Figure 5) (Wozniak et al., 2007). HSV-1 may play a role in the abnormal phosphorylation of tau proteins that then form neurofibrillary tangles and cause AD (Alvarez et al., 2012). Supportive data shows recurrent HSV-1 infections in mice result in oxidative stress in the brain (Protto, et al., 2020). HSV-1 also interferes with proteasomal degradation of damaged DNA by downregulating Ku80 protein involved in the repair of double-strand DNA breaks, eventually resulting in neuronal death (De Chiara et al., 2010).

COVID-19 has also been linked to AD. Even though it is still too early to determine the long-term harmful effects of SARS-CoV-2, several studies report signs of AD after infection, including cognitive and motor difficulties, and memory loss issues (Helms et al., 2020; Bliddal et al., 2021). Patients who experience “Long COVID” describe sleep disruption, fatigue, anxiety, depression and a “brain-fog” (inability to focus, loss of memory, and difficulty conducting normal activities) that can persist for months (Venkataramani and Winkler, 2022). SARS-CoV-2, like HSV-1, induces neuroinflammation by either direct invasion into the CNS or through peripheral infection causing systemic inflammation (De Vlieger et al., 2022). Yang et al. (2021) reported that the gene expression pattern of the microglia subpopulation induced by COVID-19 overlaps with that of microglia seen in AD patients. In addition, increased levels of the AD biomarkers Aβ, T-tau, P-tau, and neurogranin were significantly increased in the EV of COVID-infected patients (De Vlieger et al., 2022). The fact that Aβ40 and 42 levels and P-tau are increased in the EV suggests that SARS-CoV-2 contributes to the accumulation of Aβ in infected patients as well as phosphorylation of tau proteins, thus increasing the risk of developing AD (Sun et al., 2021). Brain imaging data from a UK brain-bank of pre and post COVID infection of patients showed reductions in global brain size, thickness of grey matter, and cognitive function, plus damage to olfactory tissue, indicating that long COVID accelerates the onset of AD and can exacerbate the key symptoms (Douaud et al., 2022).

Vaccinations have also been shown to reduce the risk of AD (Wu et al., 2022). Both influenza and pneumonia vaccinations reduce the risk of AD (Lehrer, 2022), with a 46-month follow up study showing that influenza vaccination decreased the risk of AD by 3.4% (Bukhbinder et al., 2022). Similarly, a recent meta-analysis indicates vaccinations against Tdap (tetanus, diphtheria, and pertussis), influenza, hepatitis A and B, and typhoid) reduce the risk for dementia (Wu et al., 2022). Other herpetic vaccinations, for instance for shingles (varicella-zoster) have been associated with a 15% reduced risk of AD and other neurodegenerative diseases (Lehrer and Rheinstein, 2022). A prospective study (albeit proof of concept with n = 49) with the *Bacillus* Calmette–Guérin (BCG) vaccine for tuberculosis has also provided positive data that will stimulate additional interest in the role of vaccines as a protection against AD (Dow et al., 2022). Whether benefits are seen with COVID-19 vaccinations remains to be analysed.

Acyclovir, a first-line treatment for HSV-1 infection, has been shown to reduce tau phosphorylation and Aβ deposition in HSV-1 infected Vero cells (Wozniak et al., 2011). Hui et al. (2020) found the combination of acyclovir and dexamethasone protective against cognitive impairment in mice injected with Aβ. Finally, vaccines against common viral infections associated with increasing the risk of AD have been developed and tested (Lehrer and Rheinstein, 2022).

## 4 Summary and conclusion

Despite the availability of drugs that target the different pathways contributing to the development of AD, the problem remains that the pre-clinical changes in brain pathology can occur decades before neuronal dysfunction and neurodegeneration become evident (Jack et al., 2010). Furthermore, prior to the introduction of the anti-amyloid MABs, the drugs available to treat AD (AChEIs and the NMDAR modulator, memantine) only provide symptomatic relief without proven efficacy to slow or reverse progression. Worse, such drugs may be ineffective if prescribed at the wrong stage of the disease and reduce compliance as a result of drug-related side-effects (Buchhave et al., 2012). Early diagnosis of MCI and AD is therefore essential so that timely intervention can be initiated, including the reduction of modifiable risk factors with appropriate therapeutic intervention, or lifestyle modifications that may also reduce the risk of unnecessary drug-specific side effects, to allow patients and families time to better plan their futures (Rasmussen and Langerman, 2019).

The approval of the anti-Aβ MABs abucanumab in June 2021, and lecanemab in July 2023 raised hopes that a magic bullet directed at the cause of the disease was now available, rather than, as for AChEIs and NMDAR modulators, merely the symptoms of AD. Although the link between familial AD and Aβ plaques is accepted anti-Aβ MABs are unlikely to benefit the majority of subjects with AD. On the positive side the data from the CLARITY trials indicated that lecanemab delayed cognitive decline inferring an approximate 6-month extension in the quality of life for patients with mild-to-moderate AD, however, there are concerns over long term use and potentially serious side-effects linked to the frequency of ARIAs. Thus, qualified conclusions about the impact of anti-Aβ MABs will require more and longer clinical trials and several years of use by ethnically diverse groups of patients with AD. Questions have also been raised as whether the modest benefits will preclude use after considering the costs of treatment and related assessments, such as frequent MRI assessment and CSF tests, and the need for a highly vigilant post-marketing surveillance (Watt et al., 2023). Ito et al. (2021) provided an estimated annual cost of drug therapy at $16,000 USDA in the US; however, that was based on 2021 costs prior to the introduction of the MABs, and the cost will vary from country to country. Collectively, these issues support arguments that although we can accept that Aβ plaques are neurotoxic it does not necessarily follow that the amyloid cascade should be accepted as dogma and causality (Herrup, 2022), and alternative hypotheses and therapeutic targets need to be vigorously pursued (Tse and Herrup, 2017).

The role of systemic inflammation as an important contributory factor to the initiation of neuroinflammation and AD deserves greater attention including the targeting of reactive microglia (Heneka et al., 2015; Lee V. M. et al., 2021). Early diagnosis together with combination therapy that includes an anti-inflammatory preferably selective for microglia would reduce neuroinflammation (Wang et al., 2023). As early as 1990 the potential of NSAIDs to offset the development of AD was proposed based on the lower prevalence of AD in patients with rheumatoid arthritis and their use of anti-inflammatory drugs (McGeer et al., 1990). Data from 17 retrospective studies also provided support (McGeer et al., 1996). Despite continuing interest (see Supplementary Figures S4) subsequent studies of NSAIDS have proved contradictory and controversial (see Table 2 for a summary). Furthermore, the chronic use of NSAIDs is associated with the risk of GI ulcers and hemorrhage, while both NSAIDs and coxibs also increase cardiovascular risk, especially in elderly patients (Wehling, 2014; Schjerning et al., 2020). Interest in the gut microbiota and its role in chronic systemic and neuroinflammation has stimulated research as to whether a leaky intestinal barrier may promote an inflammatory response that contributes to the development of AD (Mou et al., 2022). Interestingly, sodium oligomannate (GV-971), derived from brown algae and approved in China for AD, targets neuroinflammation triggered by gut bacteria (Wang et al., 2019; Xiao et al., 2021); however, data from additional studies are required for validation.

The repurposing of drugs approved for other diseases is an active field for exploration. As reflected in Figure 1, small molecule tyrosine kinase (TK) inhibitors originally developed for cancer, such as the multi-target TK inhibitor, ponatinib, and the Bruton kinase inhibitor, ibrutinib, have been suggested as having potential therapeutic efficacy to reduce neuroinflammation (Chen et al., 2019; Tan et al., 2019; Li et al., 2023). Encouragingly, a combination of ponatinib with the pan caspase inhibitor, emricasan, was found to target apoptosis and necroptosis, and reduce ischemia/reperfusion injury in the rat brain (Tian et al., 2018). Similarly, *in silico* Genome-Wide Association Studies (GWAS) analysis has identified dabrafenib, the B Raf kinase (TK) inhibitor originally developed for the treatment of malignant melanoma, as a candidate apoptosis inhibitor to protect against neurotoxicity and Parkinson’s Disease (Uenaka et al., 2018; Okamoto, 2019).

In conclusion, recognizing that there are multiple contributory factors including familial and life style that can result in the development of AD a “one-size fits all” approach to treatment is inappropriate and in addition to an early recognition and reduction of modifiable risk factors that would also help reduce systemic inflammation, new drug targets need to be explored and studied with a particular focus on early-stage intervention and metabolic contributions (Chakrabarti et al., 2015). Drug development, plus early detection and intervention, will be facilitated if biomarker testing for AD from blood tests can be validated. Besides p-tau (Ashton et al., 2024) other proteins including GFAP, neurofilament light (Nfl), growth differentiation factor-15, and latent-transforming growth factor beta-binding protein 2, have been reported to be potential blood bio-markers for AD (Guo et al., 2024; Qiang et al., 2024). Advances in pre-clinical models of AD are also required. Animal models, notably with rodents, have been the mainstay for drug testing and a large number of transgenic mouse models are available; however, there are significant limitations to the data obtained from the study of small animals (McKean et al., 2021). An *ex vivo* model that captures all of the features of the human pathology is required and an important advance was made when a human brain 3D organoid was generated using induced pluripotent stem cells (iPSCs) from subjects with AD (Raja et al., 2016). The use of 3D human stem cell models of AD should greatly facilitate drug discovery and development (Arber et al., 2017; Centeno et al., 2018).
