# Supplementary figures and images for: Alzheimer’s disease and its treatment–yesterday, today, and tomorrow
Source: Front Pharmacol. 2024 May 24;15:1399121. doi: 10.3389/fphar.2024.1399121 (PMC11167451; doi:10.3389/fphar.2024.1399121)

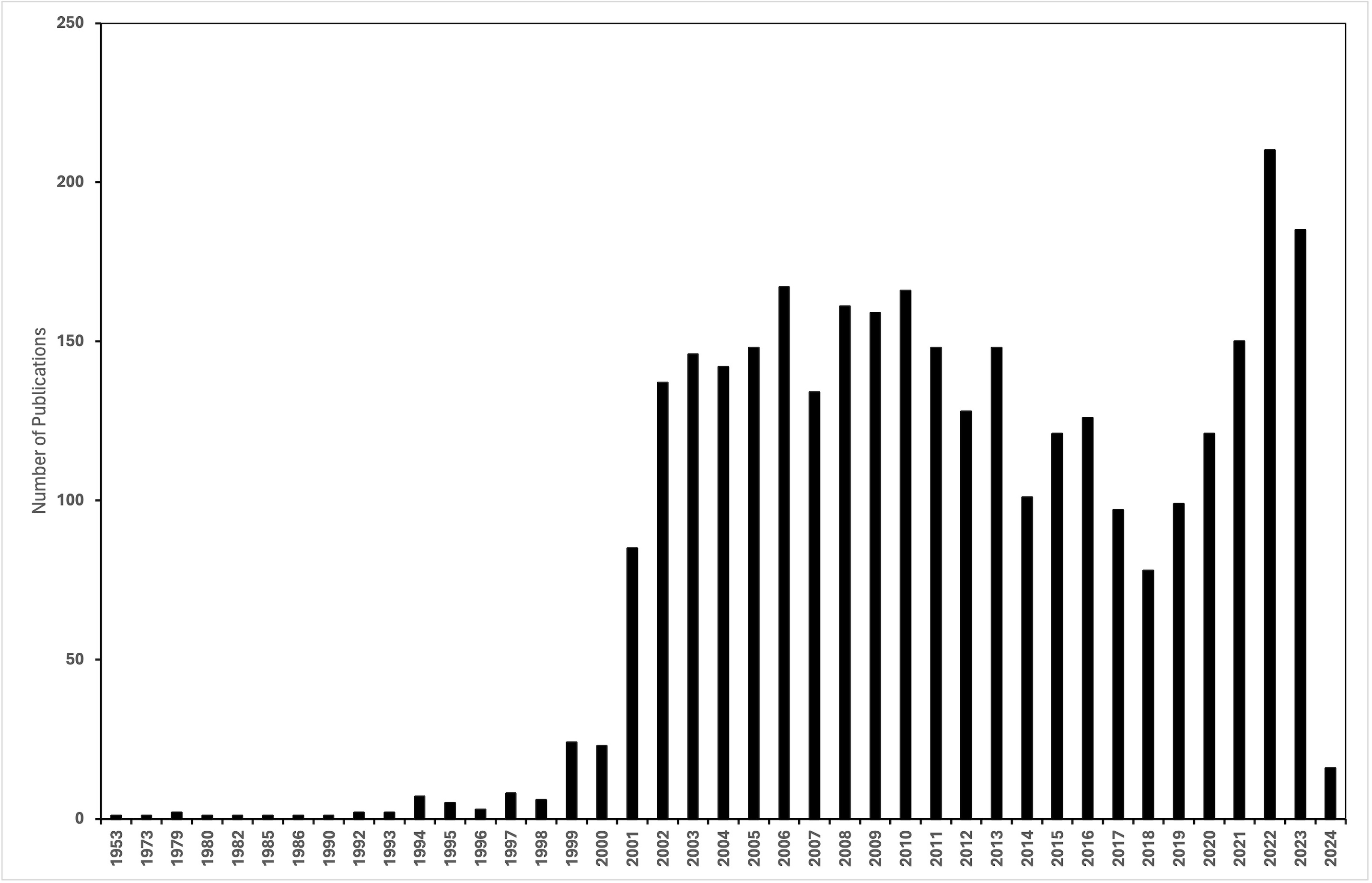

Supplement: Supplementary file 1 [file Image3.JPEG]

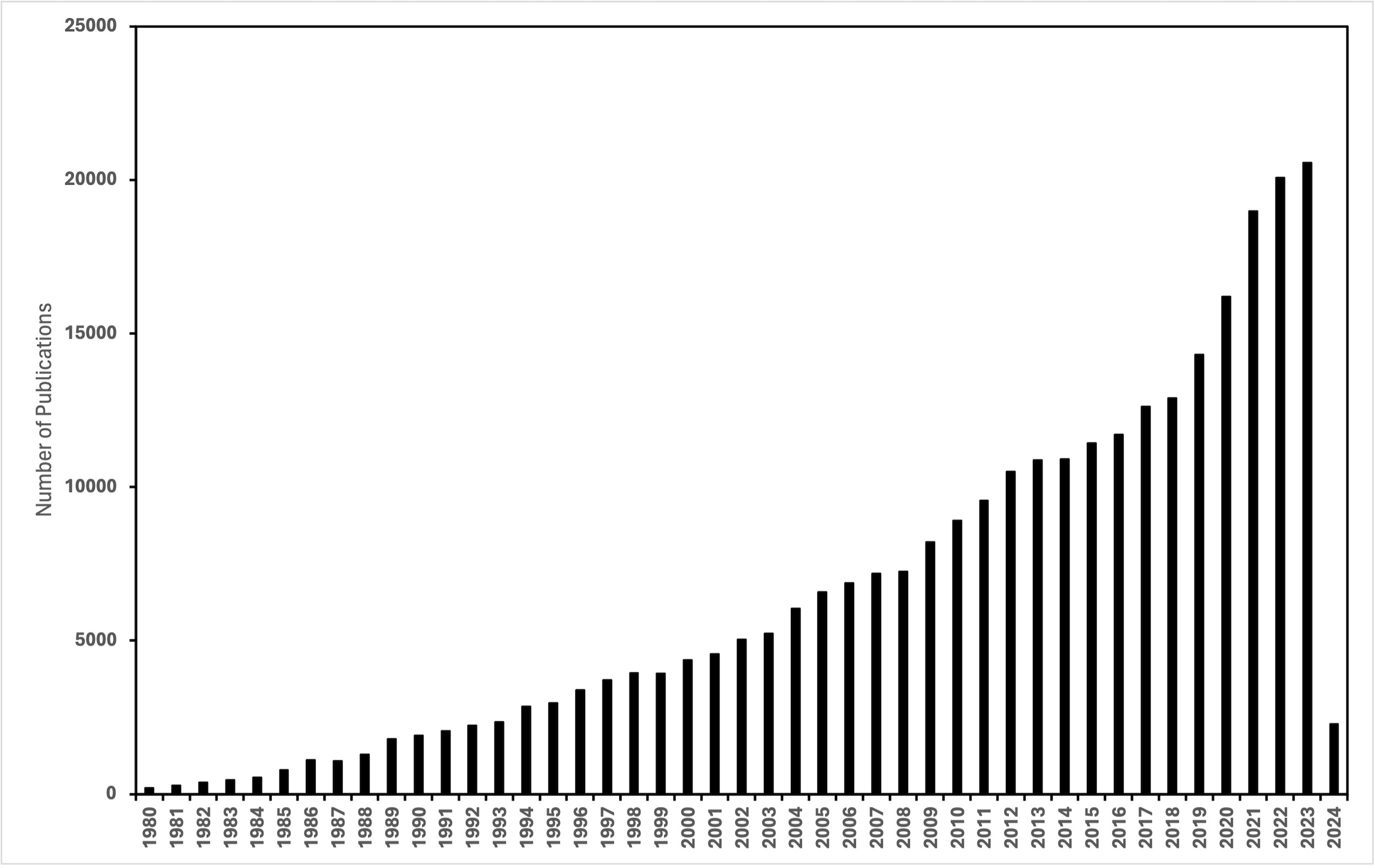

Supplement: Supplementary file 3 [file Image1.JPEG]

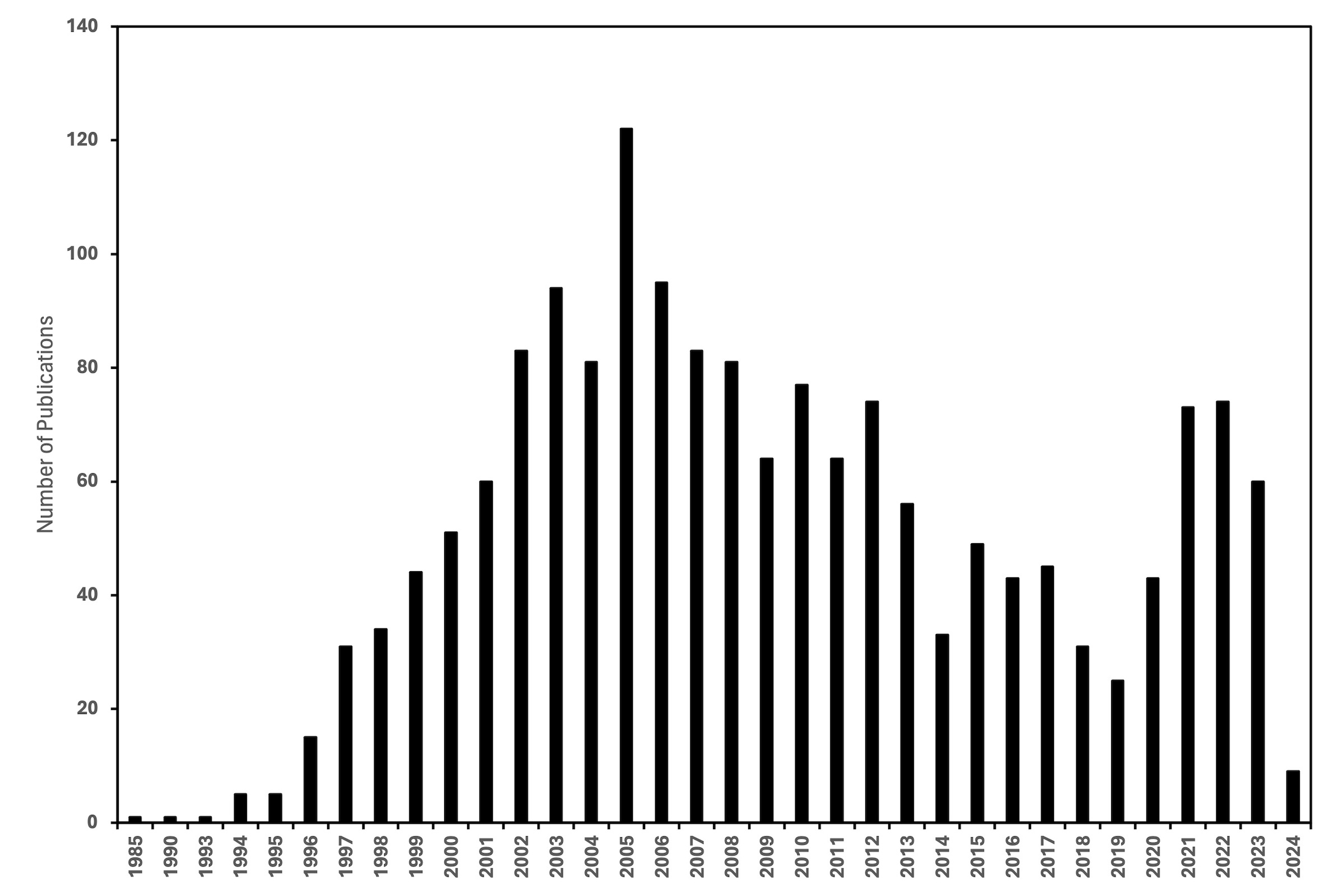

Supplement: Supplementary file 4 [file Image4.JPEG]

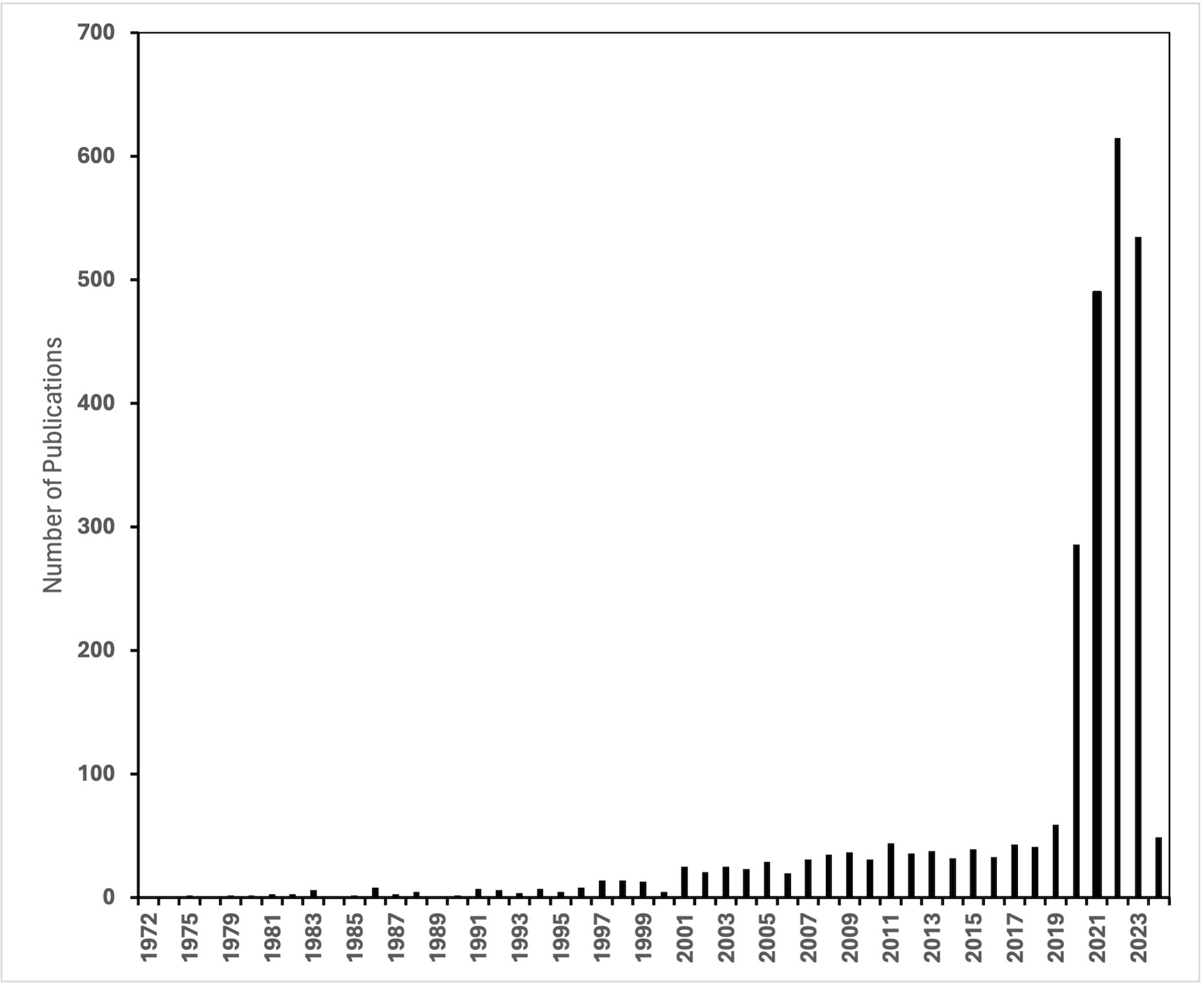

Supplement: Supplementary file 5 [file Image2.JPEG]
