# Supplementary material for: Alzheimer’s disease and its treatment–yesterday, today, and tomorrow
Source: Front Pharmacol. 2024 May 24;15:1399121. doi: 10.3389/fphar.2024.1399121 (PMC11167451; doi:10.3389/fphar.2024.1399121)
Supplement: Supplementary file 2 [file Table1.docx]

| **COGNITIVE TEST** | **DESCRIPTION** | **SCORE RANGE** | **ADVANTAGES** | **DISADVANTAGES** | **STAGE OF ALZHEIMER’S DETECTED** | **COMPONENTS TESTED** | **Key References** |
| --- | --- | --- | --- | --- | --- | --- | --- |
| **Mini-Mental State Examination (MMSE)** | Brief 30-point questionnaire to quickly assess various cognitive functions | 24-30 = normal  18-23 = MCI  0-17: moderate to severe dementia | Quick & easy.  Widely used for screening | Not sensitive enough to detect MCI or subtle deficits. | Early to moderate | Orientation to time & place.  Attention &  calculations.  Recall, language. Repetition, complex commands. | Graham et al., 2004;  Nieuwenhuis-Mark, 2010. |
| **Montreal Cognitive Assessment (MoCA)** | More comprehensive 30-point test & screening tool that assesses various cognitive domains. | 26-30 = normal  18-25 + MCI  0-17 = moderate to severe | More sensitive than MMSE for the detection of early cognitive changes. | Takes longer to administer than MMSE | Early to moderate | Memory, visuospatial ability,  Executive function, Attention and concentration. Working memory Orientation. | Dautzenberg, et al., 2020;  Jia et al., 2021; Nasreddine et al., 2005. |
| **Alzheimer’s Disease Assessment Scale-Cognitive Subscale (ADAS-Cog)** | A detailed 11-part assessment evaluating various cognitive functions.  Widely used in pharmaceutical and clinical research studies focused on AD to aid in the assessment of potential treatment before and after therapeutic interventions. | 0-11 = normal  12-19 = mild  20-35 = moderate  36-70+ = severe | Sensitive to changes in cognition and commonly used in clinical trials to track changes in cognition.  Provides more detailed evaluation of cognitive function. | Lacks sensitivity to detect MCI and is used more for patients with established AD.  More timing consuming to test patients and requires more specialized training to administer.  Not suitable for diagnosis or staging of AD. | Moderate to seal., 2016.vere | Word recall  Naming objects  Finger following commands.  Orientation  Word Recognition. | Rosen et al., 1984; Skinner et al., 2012; Podhorna et |
| **Addenbrooke's Cognitive Examination III (ACE-III)** | Updated test from ACE.  A comprehensive cognitive assessment tool that evaluates various cognitive domains.  Offers detailed profile of cognitive function, including both total and subscale scores | Total scores of:  88-100 = normal  82-87 = MCI  Below 82 = Dementia  Subscale scores:  Attention: 0-18 Memory: 0-26 Fluency: 0-14 Language: 0-26 Visuospatial: 0-16 | Covers a broad range of cognitive domains.  Sensitive to MCI and early AD.  Provides both total and subscale scores. | Administration typically takes around 15-20 minutes (Shorter than ADAS-Cog but longer than MMSE).  Variability in performance based on factors such as age, language, education, and cultural background. | Early to moderate. | Attention, memory, language, visuospatial function, and verbal fluency | Larner & Mitchell, 2014;  Beishon et al., 2019; Potts et al., 2022. |
