# Supplementary material for: Alzheimer’s disease and its treatment–yesterday, today, and tomorrow
Source: Front Pharmacol. 2024 May 24;15:1399121. doi: 10.3389/fphar.2024.1399121 (PMC11167451; doi:10.3389/fphar.2024.1399121)
Supplement: Supplementary file 6 [file Table2.docx]

| **Biomarker** | **CSF/Blood** | **Biological Significance** | **Evidence** | **Key References** |
| --- | --- | --- | --- | --- |
| **Aβ42** | Aβ42 is reduced in CSF in AD. | Aβ42, compared to variant amyloid peptides of shorter length, is primarily composed of hydrophobic peptides that tend to aggregate and sequester in plaques and deposit in brain parenchyma (Andreasen et al., 1999 | PET analysis correlates higher amyloid plaques with reduced Aβ42 in CSF and cognitive impairment. | ELISSA test Motter et al, 1995;  Blennow et al., 2015  Olsson et al., 2016; |
| **Aβ40** | Aβ40 levels are higher than Aβ42 in the CSF but show minimal to no change in AD | CSF Aβ42/Aβ40 ratio has been found to be a more accurate and sensitive as a diagnosis for AD compared to Aβ42 or Aβ40 alone, and a decrease in Aβ42/Aβ40 ratio has high concordance with amyloid PET scans | A decrease in Aβ42/Aβ40 ratio has high concordance with amyloid PET scans (Dumurgier et al., 2015; Blennow, 2018). | Portelius et al., 2007;  Olsson et al., 2016; |
| **Tau**  [Total tau (T-tau) and phosphorylated tau (P-tau)]. | (i). Elevated CSF T-tau correlates to intensity of acute neuronal damage in AD.  (ii).  p-Tau is the main component neurofibrillary tangles the formation of senile plaques. CSF P-tau levels remain stable are unchanged in neurodegenerative conditions with marked neurodegeneration but lacking tau tangles, as observed in Creutzfeldt-Jakob disease.  (iii). plasma p-tau217 showed stronger associations with brain Aβ deposition than p-tau181 and p-tau231 | (i). T-tau is less specific for AD and seen in stroke and other neurodegenerative disorders such as Creutzfeldt-Jakob disease.  (ii). P-Tau levels in CSF correlate with neurofibrillary tangles in brain. |  | Blennow et al., 2003;  Lee et al., 2019;  Riemenschneider et al., 2003;  Tapiola et al., 2009;  Skillbäck et al., 2014.  (iii). Ashton et al., 2022, 2024.  Kandimalla et al., 2013.  Drummond., 2020. |
| **Neurogranin** | Neurogranin protein linked to LTP and is associated with the dendritic spine of neurons and changes represent synaptic loss | High CSF neurogranin associated with hippocampal atrophy. and are distinctive for AD versus other neurodegenerative disorders. | Elevated CSF neurogranin are distinctive for AD versus other neurodegenerative disorders. | DeKosky et al., 1990;  Repressa et al., 1990;  Reddy et al., 2005;  Hellwig et al., 2015;  Portelius et al., 2015;  Wellington et al., 2016. |
| **Aquaporin 4**  **(AQP4)** | AQP4 is essential for clearance of Aβ and tau via the glymphatic system | High levels of AQP4 in CSF associated with AD, but also seen in other dementias. |  | Bergström et al 2021;  Arighi et al., 2022. |
